# 3-Amino-5-(indol-3-yl)methylene-4-oxo-2-thioxothiazolidine Derivatives as Antimicrobial Agents: Synthesis, Computational and Biological Evaluation

**DOI:** 10.3390/ph13090229

**Published:** 2020-09-01

**Authors:** Volodymyr Horishny, Victor Kartsev, Vasyl Matiychuk, Athina Geronikaki, Petrou Anthi, Pavel Pogodin, Vladimir Poroikov, Marija Ivanov, Marina Kostic, Marina D. Soković, Phaedra Eleftheriou

**Affiliations:** 1 Department of Chemistry, Danylo Halytsky Lviv National Medical University, Pekarska 69, 79010 Lviv, Ukraine; vgor58@ukr.net; 2 InterBioScreen, 142432 Chernogolovka, Moscow Region, Russia; vkartsev@ibscreen.chg.ru; 3 Department of Chemistry, Ivan Franko National University of Lviv, Kyryla i Mefodia 6, 79005 Lviv, Ukraine; v_matiychuk@ukr.net; 4 School of Pharmacy, Aristotle University of Thessaloniki, 54124 Thessaloniki, Greece; anthi.petrou.thessaloniki1@gmail.com; 5 Institute of Biomedical Chemistry, Pogodinskaya Street 10 Bldg.8, 119121 Moscow, Russia; pogodinpv@gmail.com (P.P.); vladimir.poroikov@ibmc.msk.ru (V.P.); 6 Mycological Laboratory, Department of Plant Physiology, Institute for Biological Research, Siniša, Stanković-National Institute of Republic of Serbia, University of Belgrade, Bulevar Despota Stefana 142, 11000 Belgrade, Serbia; marija.smiljkovic@ibiss.bg.ac.rs (M.I.); marina.kostic@ibiss.bg.ac.rs (M.K.); mris@ibiss.bg.ac.rs (M.D.S.); 7 Department of Biomedical Sciences, School of Health Sciences, International Hellenic University, Sindos, 57400 Thessaloniki, Greece; eleftheriouphaedra@gmail.com

**Keywords:** indole, thioxothiazolidine, antibacterial activity, antifungal activity, computer-aided prediction, docking, Mur B, CYP 51

## Abstract

Herein we report the design, synthesis, computational, and experimental evaluation of the antimicrobial activity of fourteen new 3-amino-5-(indol-3-yl) methylene-4-oxo-2-thioxothiazolidine derivatives. The structures were designed, and their antimicrobial activity and toxicity were predicted in silico. All synthesized compounds exhibited antibacterial activity against eight Gram-positive and Gram-negative bacteria. Their activity exceeded those of ampicillin and (for the majority of compounds) streptomycin. The most sensitive bacterium was *S. aureus* (American Type Culture Collection ATCC 6538), while *L. monocytogenes* (NCTC 7973) was the most resistant. The best antibacterial activity was observed for compound **5d** (Z)-N-(5-((1H-indol-3-yl)methylene)-4-oxo-2-thioxothiazolidin-3-yl)-4-hydroxybenzamide (Minimal inhibitory concentration, MIC at 37.9–113.8 μM, and Minimal bactericidal concentration MBC at 57.8–118.3 μM). Three most active compounds **5d, 5g,** and **5k** being evaluated against three resistant strains, Methicillin resistant *Staphilococcus aureus* (MRSA), *P. aeruginosa,* and *E. coli*, were more potent against MRSA than ampicillin (MIC at 248–372 μM, MBC at 372–1240 μM). At the same time, streptomycin (MIC at 43–172 μM, MBC at 86–344 μM) did not show bactericidal activity at all. The compound **5d** was also more active than ampicillin towards resistant *P. aeruginosa* strain. Antifungal activity of all compounds exceeded those of the reference antifungal agents bifonazole (MIC at 480–640 μM, and MFC at 640–800 μM) and ketoconazole (MIC 285–475 μM and MFC 380–950 μM). The best activity was exhibited by compound **5g**. The most sensitive fungal was *T. viride* (IAM 5061), while *A. fumigatus* (human isolate*)* was the most resistant. Low cytotoxicity against HEK-293 human embryonic kidney cell line and reasonable selectivity indices were shown for the most active compounds **5d**, **5g**, **5k**, **7c** using thiazolyl blue tetrazolium bromide MTT assay. The docking studies indicated a probable involvement of *E. coli* Mur B inhibition in the antibacterial action, while CYP51 inhibition is likely responsible for the antifungal activity of the tested compounds.

## 1. Introduction

Infectious diseases affect large populations and cause significant morbidity and mortality [1]. They represent a global indirect load on public health security and an impact on socio-economic stability worldwide. Bacterial, fungal, and viral infections have monopolized the dominant factors of death and disability of millions of humans for centuries. They are presently plaguing and even ravaging populations worldwide each year with performances far surpassing wars [2].

It should be mentioned that several dozen new infections have grown and affected the health of billions of people over the world, mainly in developing countries [3]. Unfortunately, there are no successful pharmaceuticals or vaccines for many of these new infections [3].

The treatment of infectious disease is still an imperative and demanding problem due to the growing number of multi-drug resistant pathogens, especially Gram-positive bacteria. Due to this, the lack of effective antimicrobial drugs, morbidity, and mortality notably increased [4].

Drug resistance causes vast human suffering, and now it is one of the most significant challenges of the twenty-first century. Species such as the methicillin-resistant *S. aureus* and vancomycin-resistant enterococci have emerged due to the irrational or overuse of antimicrobial agents [5].

The pathogens, including *Enterococcus faecium*, *Staphylococcus aureus*, *Klebsiella pneumoniae*, *Acinetobacter baumannii*, *Pseudomonas aeruginosa*, and *Enterobacter spp*. which also called ESCAPE pathogens, are of particular importance since they play a significant role affecting several human organs including the lung and urinary system. Besides, they exhibited increased resistance to clinically used antibiotics [6].

Numerous of these pathogens are Gram-negative bacteria, which are of specific concern due to their resistance of up to 50% against carbapenems that have been reported in some developing countries [6]. Despite the availability of some new antibiotics against Gram-positive pathogens, no treatment of these pathogens with a new class of compounds has been introduced in the last 40 years. Therefore, to overcome the resistance, the discovery of safer and more effective antimicrobial agents with a different mechanism of action is still an urgent need [7].

The interest in thiazolidine-based compounds attracted the attention of medicinal chemists, and a plethora of them have been studied to evaluate pharmacological properties [8,9,10]. Despite the appearance of some controversial opinions regarding the analysis of the molecular mechanism of their action, prominent representatives among the developed drug-like molecules are thiazolidinone derivatives [11,12] since they are a valuable source of building blocks for the development of novel molecules [13,14,15].

N-(4-oxo-2-thioxothiazolidin-3-yl)carboxamides exhibit antimicrobial [16,17,18,19,20] and antitumor [21,22,23] actions, are dual COX-1/2 and 5-LOX inhibitors [24,25], non-nucleoside inhibitors of Hepatitis C NS5b RNA polymerase [26,27] and HIV-1 reverse transcriptase inhibitors [28].

The combination of the thiazolidinone ring with other pharmacologically promising heterocycles has been a warranted approach for developing new “drug-like” molecules with the desired activity profile [29,30,31]. Our previous studies showed that thiazolidinone core with indole fragment in one molecule gave the compounds with high antimicrobial activity [19].

**Figure 1:**
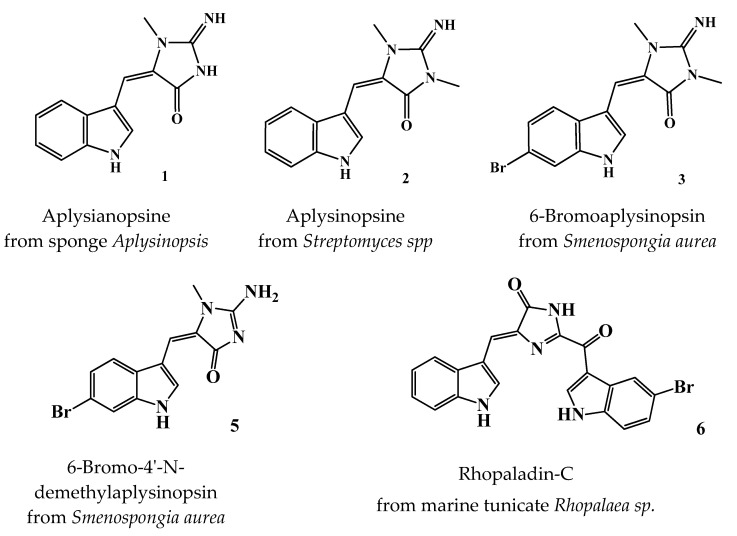
Structure of indole-containing azahydantoins 1-5 from sponges and streptomycetes.

**Figure 2:**
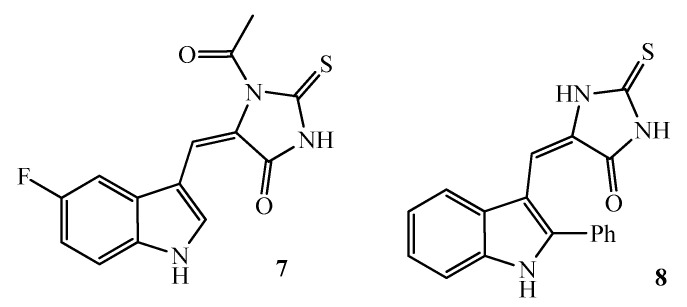
Synthetic thiohydantoin analogues.

On the other hand, indole derivatives represent another scaffold widely spread in nature with a broad spectrum of biological activities. The indole ring was found not only in natural compounds but also in diverse semisynthetic and synthetic drug-like molecules [32,33]. They exhibit antimicrobial [34,35,36,37,38,39], anti-inflammatory [40,41], COX inhibitory [42,43] anticancer [44,45,46], antiviral [47,48], anti-HIV [49,50], and antidiabetic [51] activities. Among the natural compounds containing the indolene fragment, several imidazoline and imidazolidine alkaloids are known, which have a wide spectrum of biological activity, including antibacterial. Thus, indole-containing azahydantoins 1-6 from sponges and streptomycetes have a potent antibacterial and antiseptic action (Figure 1) [52,53,54]:

It is also known that synthetic thiohydantoin (rhodanine) analogs **7**, **8 (**Figure 2**)**, exhibit pronounced antibacterial properties [55].

Therefore, the design and development of hybrid molecules combining thiazolidinone and indole cores in the same structure is a promising approach. Taking into account all issues mentioned above and encouraging results obtained in our earlier studies [19], in this paper, we present the synthesis and biological evaluation of new (1H-indole-3-yl-methylene)-4-oxo-2-thioxothiazolidin derivatives with potent antimicrobial activity.

## 2. Results and Discussion

### 2.1. In Silico Antimicrobial Activity Estimation

#### 2.1.1. Antibacterial Activity

Using AntiBac-Pred [56] one of the predictive web services of Way2Drug platform [57], activity against at least one strain of bacteria was predicted for each of the fourteen designed compounds with Pa-Pi values in the range from 0.001 to 0.309. According to the prediction results, the highest probability of antibacterial activity against the *Bacillus subtilis subsp. subtilis* str. 168 was estimated for derivatives **7a** and **5b** (Pa-Pi values are 0.309 and 0.305, respectively).

Similarly, we estimated in silico the probability of antibacterial activity for the reference drugs streptomycin and ampicillin. For both reference drugs, wide antibacterial action was predicted. For the top-10 predictions of streptomycin Pa-Pi values vary from 0.905 to 0.947; for ampicillin—from 0.712 to 0.989. Contrary, for relatively new antibacterial agent trifolirhizin, which structure was disclosed only on July 7, 2020 (Clarivate Analytics Integrity [58]), the top-10 predictions Pa-Pi values vary from 0.369 to 0.552.

#### 2.1.2. Antifungal Activity

Using web service AntiFun-Pred [59], activity against at least one of the fungal species was predicted for six of the fourteen studied compounds with Pa-Pi values ranging from 0.001 to 0.112. The results show that among the studied compounds, derivatives 5a (Pa-Pi against *Trichophyton mentagrophytes* equals 0.112) and 7a (Pa-Pi against *Candida equals* 0.101) have better chances to be found active in biological evaluation of the antifungal activity.

The results of in silico antimicrobial activity assessment are given in the supplementary file PASSweb_results_13mols.xlsx. Small Pa-Pi values reflect the novelty of the analyzed compounds compared to those included in the PASS training set.

Similarly, for the reference drug ketoconazole wide antifungal action was predicted with Pa-Pi values in the range 0.622–0.812 (top-10 predictions), while for the new antifungal agent drimenin disclosed on 12 June 2020 (Clarivate Analytics Integrity [58]), only two antifungal activity were predicted with Pa-Pi values 0.007 and 0.030.

#### 2.1.3. Acute Rat Toxicity 

Using web service based on GUSAR software [60,61], acute rat toxicity with regards to different administration routes was estimated for the studied compounds. LD50 values and toxicity classes are given in Table 1. Most of the predictions indicate that the studied compounds belong to the fifth or fourth rodent toxicity classes.

### 2.2. Chemistry

**Scheme 1:**
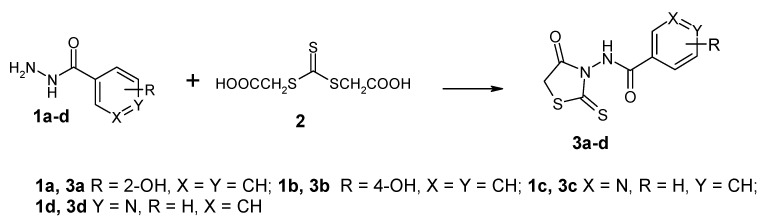
Synthesis of initial compounds.

The starting N-(4-oxo-2-thioxothiazolidin-3-yl) -carbamides **3a-d** was prepared by reacting the acid hydrazides **1a-d** with trithiocarbonyl diglycolic acid (Scheme 1). The reaction was carried out in a medium of boiling aqueous alcohol. The yield of the products was 83–97%.

**Scheme 2:**
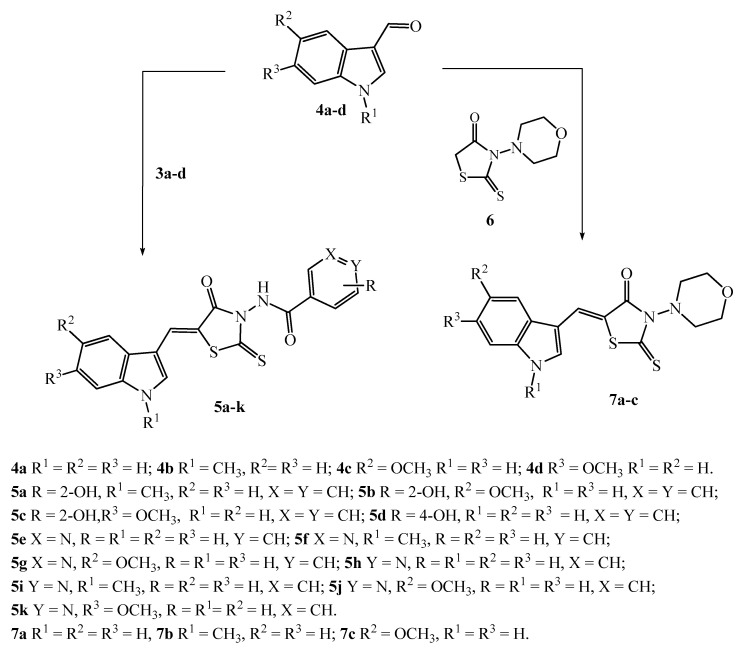
Synthesis of final compounds.

The titled compounds were synthesized according to the process shown in Scheme 2.

The reaction of N-(4-oxo-2-thioxothiazolidin-3-yl)carbamides **3a–d** with indole-3-carbaldehydes **4a-d** in acetic acid in the presence of an ammonium acetate catalyst afforded with high yield 5-[(R-1*H*-indol-3-yl)methylene]-4-oxo-2-thioxothiazolidin-3-ylcarbamides **5a–k**, while upon reaction of indole-3-carbaldehydes **4a–d** with 3-morpholino-2-thioxothiazolidin-4-one **6** in the same conditions 5-[(R-1*H*-indol-3-yl) methylene] -3-morpholin-4-yl-2-thioxothiazolidin-4-ones **7a–c** were obtained.

All compounds were characterized by IR, ^1^H and ^13^C NMR spectroscopy. In the IR spectra of compounds **3a–d, 5a–k,** and **7a, 7c**, the carbonyl group of the 4-thiazolidone ring absorbs at 1753.21–1690.53 cm^−1^, and the thiocarbonyl group—at 1608.56–1556.48 cm^−1^. The absorption band of the carbonyl group of the amide fragment of **3a–d** and **5a–k** is located at 1689.56–1654.84 cm^−1^.

In the starting 3-substituted 2-thione-4-thiazolidones, the amide proton NH-CO of the compounds **3a–d** appears as a singlet in the range 11.95-10.91 ppm, and the cyclic methylene group resonates as a singlet or quartet at 4.55–4.48 ppm. etc. In the target products **5a–k**, the amide proton is in the range of 11.85–11.12 ppm. The 5-methylidene proton CH = of compounds **5a–k** and **7a, 7c** resonates in the form of a singlet at 8.20–7.94 ppm, which, according to the literature [9,62], is characteristic of the Z isomer. The singlet NH of the protons of the indole ring appeared in the range 12.31–12.06 ppm.

### 2.3. Biological Evaluation

#### 2.3.1. Antibacterial Activity 

Compounds **5a–k** and **7a–c** were evaluated for antibacterial activity, by microdilution method to determine the minimal bacteriostatic and bactericidal concentrations. As reference compounds, we used ampicillin and streptomycin, which are both broad-spectrum antibiotics commonly applied to treat different conditions. Antibacterial activity of tested compounds is shown in Table 2 with MIC values in the range of 36.5–211.5 µM and MBC at 73.3–282.0 µM. According to the order of activity which can be presented as: **5d** > **5g** > **5k** > **5j** > **5c** > **5h** > **5e** > **5f** > **5a** > **7c** > **7b** > **5b** > **7a** > **5i** the best activity is achieved for compound **5d** with MIC at 37.9–113.8 µM and MBC at 75.9–151.7 µM. The lowest antibacterial activity was observed for compound **5i** with MIC values in the range of 76.1–152.1 µM and MBC at 152.1–304.2 µM. The most sensitive bacterium appeared to be *S.aureus* (ATCC 6538), *En. cloacae* (ATCC 35,030) was the second most sensitive, while *S.Typhimirium* was the most resistant one. Another resistant strain was Gram-negative bacterium *S. Typhimurium* (ATCC 13,311).

Compound **7b** exhibited good activity against *B. cereus* with MIC and MBC at 41.7 and 83.4 µM respectively. Compound **5d** appeared to be potent against *S. aureus* (ATCC 6538), *P. aeruginosa* (ATCC 27,853), and *En. cloacaei* (ATCC 35,030) with MIC at 37.9 µM and MBC at 75.9 µM. It also showed good activity against *B. cereus* with MIC and MBC at 55.6 and 75.9 µM respectively. Compound **5h** appeared to be potent against *En. cloacae* and *P. aeruginosa* (ATCC 27,853) with MIC and MBC at 39.4 and 78.9 µM. Good activity against these two species and *S.* aureus (ATCC 6538) was also shown by compound 5j (MIC/MBC 58.6/73.1 µM). Good activity against *S. aureus* (ATCC 6538)*,* also exhibited by compound **7b** with MIC at 41.7 µM and MBC at 83.4 µM. On the other hand, compound **5g** exhibited good activity against *En. cloacae* (ATCC 35030)*, S. aureus* (ATCC 6538), and *S. typhimurium* (ATCC 13311) with MIC/MBC values 36.5/73.1 and 53.6/73.1 µM, respectively. It is worth to notice that all compounds appeared to be more potent than ampicillin against all bacteria used and more active than streptomycin against all bacteria except *B. cereus* and *S. typhimurium* (ATCC 13,311).

The structure-activity studies revealed that the most beneficial for antibacterial activity is the presence of hydroxybenzamide (**5d**) on the N-atom of (Z)-5-((5-methoxy-1H-indol-3-yl)methylene)-3-morpholino-2-thioxothiazolidin-4-one. Introduction of the 5-methoxy group to indole ring and replacement of hydroxybenzamide by nicotinamide (**5g**) decreased a little activity while shifting of methoxy group from position 5 to position 6 of indole ring and replacement of nicotinamide by isonicotinamide led to less active compound **5k** compared to compound **5g**.

On the other hand, the isonicotinamide derivative of (Z)-5-((1-methyl-indol-3-yl)methylene)-2-thioxothiazolidin-4-one **(5i)** appeared to be the less active compound. It was observed that for (Z)-5-[(1H-indol-3-yl)methylene]-2-thioxothiazolidin-4-one (**5h**) as well as for (Z)-N-5-[(1-methyl-1H-indol-3-yl)methylene]-4-oxo-2-thioxothiazolidin-4-one (**5g**) derivatives isonicotinamide substituent is endowed with better activity. The opposite was observed for 6-methoxy indole derivatives where more preferable is nicotinamide as a substituent (**5k**). Between methylindole derivatives (**5a, 5f, 5i**), more favorable for activity was nicotinamide substituent (**5f**), followed by benzamide, (**5a**) while isonicotinamide (**5i**) had a negative effect on antibacterial activity. For -2-hydroxybenzamides derivatives more preferable for antibacterial activity appeared to be 6-methoxy substitution of indole ring (**5c**) followed by methylidole (**5a**) while 5-methoxy substitution on indole ring was negative leading to one of the less active compounds **(5b**). In the case of 3-morpholino-2-thioxothiazolidin-4-one derivatives (**7a–c**), which were among the less active compound, it seems that 5-methoxy substitution on indole ring is preferable than methylindole or indole ring.

Thus, it can be concluded that the most favorable effect on the antibacterial activity of the target compounds is provided by the introduction into the molecule of an unsubstituted indolidene and 6-methoxyindolidene fragment. In addition, the nature of the substituent at position 3 of the thiazolidine ring has a direct influence on the enhancement of the antibacterial action. An increase in the antibacterial effect is observed from the use of 4-hydroxybenzamide and isonicotinamide substitutes.

From all mentioned above, it is evident that the antibacterial activity of these compounds depends not only on substituent and its position in the indole ring but also on substituent on the N-atom of 2-thioxothiazolidin-4-one ring.

Three most active compounds were also evaluated against the resistant strains, including MRSA, *P. aeruginosa*, and *E. coli*, (Table 3). From the obtained results, it is evident that all three compounds were more active against MRSA than ampicillin, while streptomycin did not show any bactericidal activity. The compound **5d** was also more active than ampicillin towards resistant *P. aeruginosa* strain.

#### 2.3.2. Antifungal Activity

All compounds also showed antifungal activity with MIC values ranging from 9.7 to 347.4 μM and MFC at 19.5–694.8 μM.The antifungal activity of compounds is shown in Table 4 and follows the order: **5g > 7c > 7b > 5d > 5b > 5e > 5k > 5f > 5j > 5c > 5i > 5a > 7a > 5h**. Compound **5g** displayed the best activity with MIC values in the range of 9.7–73.1 μM and MFC at 36.5–146.2 μM, while compound **5h** exhibited the lowest potential with MIC and MFC at 28.9–315.5 μM and 39.4–630.9 μM respectively. It was observed that similar to bacteria, fungi showed different sensitivity towards compounds tested. Thus, the most sensitive fungal strain appeared to be *T. viride* (IAM 5061), while the most resistant filamentous A. fumigatus. The behavior of compounds towards fungi species was different, too.

Several compounds showed very good activity against some species. For example, compound **5d** exhibited good activity against the most resistant *A. fumigatus* (MIC/MFC at .20.2/37.9 μM, while compound **7b** against *T. viride* (IAM 5061), *P. cyclpoium var verucosum* (food isolate) and all Aspergillus species except *A. fumigatus* (human isolate) with MIC at 22.3 μM and MFC at 41.4 μM. Compound **5g** exhibited excellent activity against *T. viride* (IAM 5061) (MIC/MFC at 0.97/1.95 µmol/mL × 10^−2^). Additionally, good activity was achieved for compound **5g** against *A. versicolor* (ATCC 11730), *A. ochraceus* (ATCC 12066), *P. funiculosum* (ATCC 36839) with MIC and MFC at 19.5 μM and 36.5 μM respectively. Compound **5c** appeared to be potent against *A. ochraceus* (ATCC 12066) and *T. viride* (IAM 5061) (MIC/MFC at 18.8/35.3 μM whereas compound **7c** exhibited very good activity against *T. viride* (IAM 5061)with MIC at 10.7 µM and MFC at 21.3 µM and also good activity against *A. ochraceus* (ATCC 12066) and *P. funiculosum* (ATCC 36839 (MC/MFC at 23.1/39.9 µM. The potential of ketoconazole was at MIC 285-475 µM and MFC at 380–950 µM. Bifonazole displayed MIC at 480-640 µM and MFC at 640–800 µM. It should be mentioned that all compounds appeared to be more potent than ketoconazole and bifonazole. Only compound **7a** against *A. fumigatus (human isolate)* was less active than bifonazole.

According to the analysis of the structure-activity relationships, the most beneficial for antifungal activity is the presence of the 5-methoxy group in indole ring as well as nicotinamide as a substituent of the side chain (**5g**). In contrast, the presence of isonicotinamide in methylindole (**5i)** derivative appeared to be detrimental. Shifting of 5-OMe of compound **5g** to position 6 of indole and replacement of nicotinamide by 2-hydroxybenzamide resulted in compound **5c** with decreased activity. Removal of methoxy group and introduction of morpholino moiety to the N atom of thioxothiazolidinone **(7a)** decreased more activity. 

In indole derivatives (**5d**, **5e**, **5h**), the presence of 4-hydroxybenzamide was favorable for antifungal activity, while isonicotinamide substituent had a negative effect. On the contrary, for methylindole derivatives (**5a**, **5f**, **5i**), the negative impact was observed with the presence of 2-hydroxybenzamide, while in the case of the 5-methoxy indole derivatives (**5b**, **5j**) it was the opposite. Finally, for the derivatives with morpholino moiety, the best activity was observed with the presence of the 5-methoxy group in the indole ring. The indole derivative was one of the less potent.

Thus, as in the case of antibacterial activity, antifungal activity depends not only on substitution in the indole ring but also on substituent on the N-atom of the 2-thioxothiazolidinone ring. In the series of (Z)-5-((5-methoxy-1H-indol-3-yl)methylene)-3-morpholino-2-thioxothiazolidin-4-one derivatives the most important structural features which enhanced the antifungal activity are again 4-hydroxybenzamide and 1H-indole moiety as well as nicotinamide and 5- and 6-methoxyindole moieties. On the other hand, in the series of indole 3-methylene morpholino-2-thioxothiazolidin-4-one derivatives, the presence of the 5-OCH3 group in the indole ring enhance the antifungal activity.

#### 2.3.3. Cytotoxicity Assessment

Low toxicity and selectivity of action of antimicrobial compounds is a crucial pre-requisite for further development. Thus, we studied the cytotoxicity of the most active compounds. MTT analysis was performed on the HEK-293 human embryonic kidney cell line. The cells were cultured in DMEM medium supplemented with 10% fetal bovine serum. The cells were inoculated into a 96-well plate at a concentration of 5^.^10^4^/mL (5^.^10^3^ per well, 100 µL each). After one day of culture, compound preparations were added, and the results were obtained after a 72 h culture period. The compounds were added at four concentrations (25, 50, 100, and 250 µM). Since compound solutions contained DMSO, control cultures containing only DMSO at the final concentration obtained when the appropriate volume of compound solution was added were performed. 

**Figure 3:**
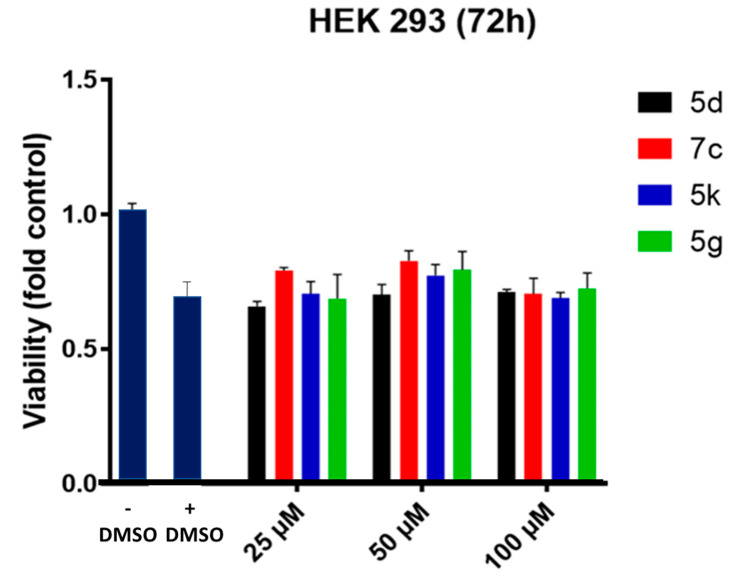
MTT assay results for compounds **5d, 5k, 5g, 7c.** According to the results, all compounds did not show statistically significant, concentration-dependent cytotoxicity at concentrations up to 100 μM. The stable decrease in viability observed can be attributed to dimethyl soulfoxide (DMSO,) present at stable concentration at all compound samples.

Although the compounds do not exhibit statistically significant concentration-dependent toxicity up to 100 µM (Figure 3), they show some toxicity at higher concentrations. The average CC_50_ values obtained from three different experiments are given in Table 5 and Table 6. The SI index is also shown in Table 5 and Table 6.

Compound **5g** and **7c** exhibited the best SI index for anti-fungal activity while compound **5d** exhibited the best SI index for anti-bacterial activity. 

We compared the CC_50_ values of compounds **5d**, **5k**, **5g**, **7c** with cytotoxicity of the reference drugs obtained in the HEK-293 human embryonic kidney cell line. For antibacterials streptomycin, ampicillin and antifungal bifonazole CC_50_ exceeded 100 µM [63,64]; for antifungal ketoconazole CC_50_ = 60 µM [65]. Thus, cytotoxicity of the most active compounds in our study is comparable or lower than cytotoxicity of the reference antimicrobial drugs.

### 2.4. Docking Studies

Since the mechanism of antimicrobial action of our compounds is not known, to choose the proteins as potential targets, we based on the literature. It was found that benzothiazole derivatives are mentioned as Gyrase inhibitors [66,67,68]. On the other hand, according to the literature, thiazolidinones act as MurB inhibitors [69,70,71,72]. Furthermore, prediction of the mechanism of action by computer program PASS indicated Thymidylate kinase as the probable antibacterial target. On the other hand, several publications mentioned thiazolidinone and indole derivatives as 14^α^-lanosterol demethylase inhibitors [73,74,75]. Thus, taking all these into account, we proposed *E. coli* DNA Gyrase, Thymidylate kinase, and *E. coli* MurB enzymes as antibacterial targets, with CYP51 as the antifungal target.

#### 2.4.1. Docking to Antibacterial Targets 

The docking studies revealed that estimated binding energy to *E. coli* DNA Gyrase (−2.59 to −6.54 kcal/mol) as well as to thymidylate kinase (−1.55 to −4.12 kcal/mol), were higher than that to *E. coli* MurB (−7.07 to −10.93 kcal/mol). Therefore, it may be resolved that *E. coli* MurB is the most suitable enzyme where binding scores were consistent with biological activity (Table 7).

**Figure 4:**
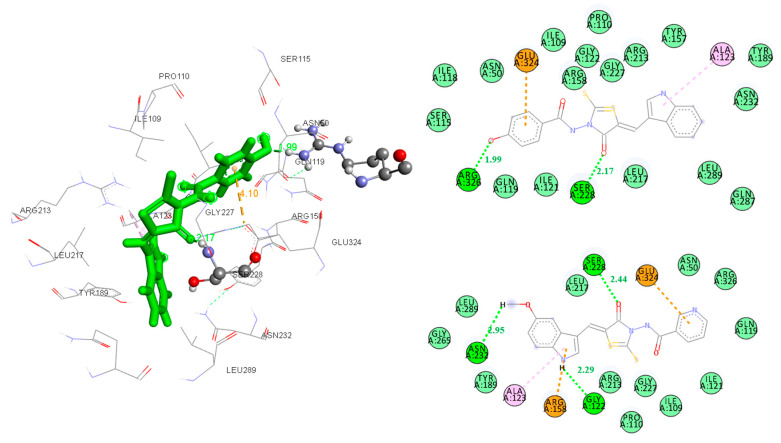
Docked conformation of the most active compound **5d** in *E.coli* MurB **(Left)**. 2D diagrams of the most active compounds **5d** (up) and **5g** (down) in *E.coli* MurB **(Right)**.

The docking pose of the most active compound **5d** in *E. coli* MurB enzyme showed two favorable hydrogen bond interactions. The first one is between the oxygen atom of the C=O group of the compound and the hydrogen of the side chain of Ser228. The second one between the oxygen atom of -OH group of the compound and the side chain of Arg326 (distances 2.17 Å and 1.99 Å, respectively). The fused rings interact hydrophobically with the residues Tyr189, Asn232, Leu289, Ala123, Leu217, and Arg213, while the benzene ring interacts hydrophobically with the residues Asn50, Ser115, Ile118, Ile121, Gln119 and Glu324 (Figure 4). These interactions stabilize the complex compound-enzyme and play a crucial role in the increased inhibitory activity of compound **5d** Moreover, the hydrogen bond formation with the residue Ser228 is essential for the inhibitory action of the compounds; thus, this residue takes part in the proton transfer at the second stage of peptidoglycan synthesis [76].

**Figure 5:**
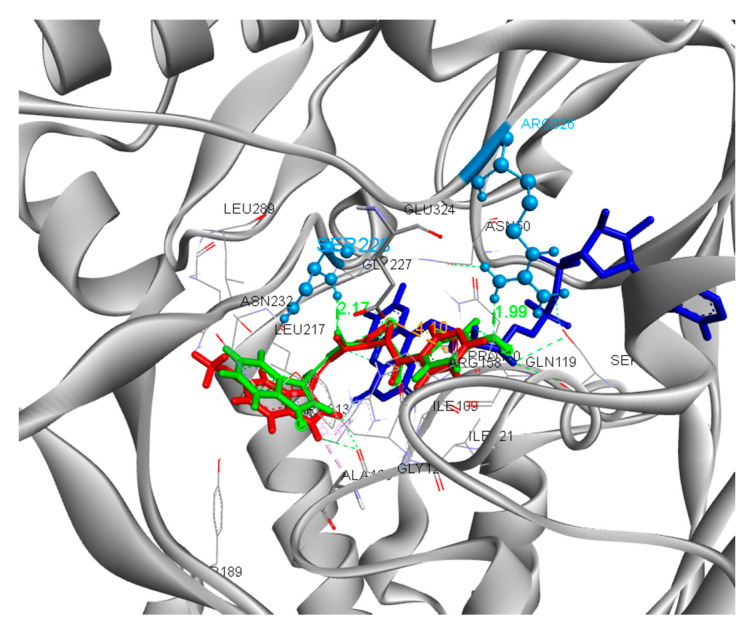
Docked conformation of compounds **5d** (green), **5g** (red) and FAD (blue) in *E.Coli* MurB.

The second most active compound, **5g,** also forms the hydrogen bond interaction with the residue Ser228 that explains its high inhibitory action (Figure 2). Detailed analysis of the docking pose of the two most active compounds showed that they similarly bind MurB, and they insert deeper to the binding center of the enzyme than FAD, forming a hydrogen bond with the residue Ser228 (Figure 5).

The same behavior was observed in the case of docking of the most active compound among 5-(1*H*-indol-3-ylmethylene)-4-oxo-2-thioxothiazolidin-3-yl)alkane carboxylic acids [19] and 5-adamantane thiadiazole-based thiazolidinones [70]. Again, the formation of the hydrogen bond between the C=O group and Ser228 was observed. Thus, the obtained results support previous data [69,70,71,72] that MurB maybe is the most appropriate target for the antibacterial activity for this chemical series.

#### 2.4.2. Docking to Lanosterol 14α-demethylase of *C. albicans*

All the synthesized compounds and the reference drug ketoconazole were docked to lanosterol 14α-demethylase of *C. albicans* (Table 8).

**Figure 6:**
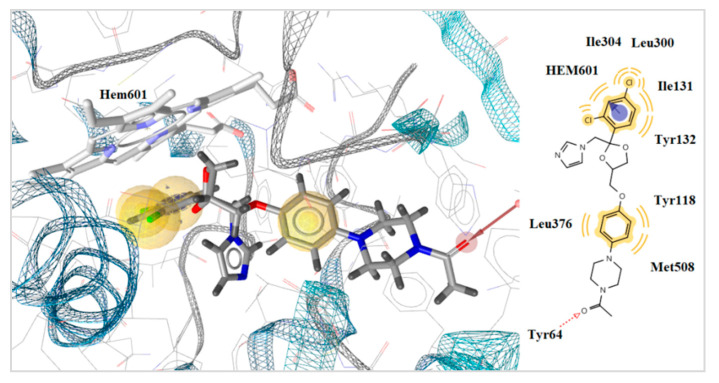
Docked conformation of ketoconazole in lanosterol 14alpha-demethylase of *C*. *albicans* (CYP51_ca_).

**Figure 7:**
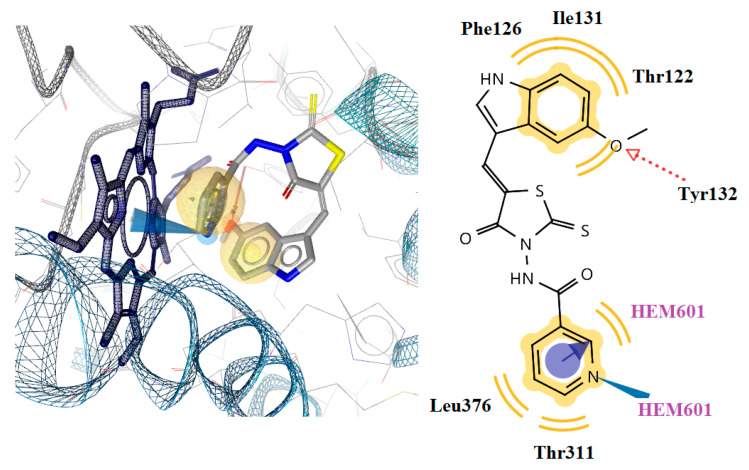
Docked conformation of compound **5g** in lanosterol 14alpha-demethylase of *C*. *albicans* (CYP51_ca_).

Docking results showed that all the synthesized compounds might bind to CYP51_Ca_ close to those of the reference drug ketoconazole. Compound **5g** is located inside the enzyme alongside to heme group, interacting with the Fe of the heme group of CYP51_Ca_ throughout its atom N of the pyridine ring. Moreover, compound **5g** forms a hydrogen bond between the oxygen of –OCH_3_ substituent and the hydrogen of the side chain of Tyr132. Hydrophobic interactions were detected between residues Thr122, Phe126, Tyr132, and Ile131 and the fused rings of the compound **5g**, also between Leu376, Thr311 and the benzene ring of the compound. Furthermore, compound **5g** interacts hydrophobically throughout its benzene ring with the heme group of the enzyme, and also it forms a positive ionizable bond with it (Figure 6). Interaction with the heme group was also observed with the benzene ring of ketoconazole, which forms positive ionizable interactions (Figure 6 and Figure 7). However, compound **5g** forms a more stable complex of the ligand with enzyme indicating its interaction with the Fe, which is probably why compound **5g** showed high antifungal activity.

It should be mentioned that the tested compounds interact more strongly with the heme group of the enzyme CYP51_Ca_ because the heme’s Fe is involved in this interaction. In the case of our previous work [19], the most active compound interacts with the heme but throughout its benzene ring and the –NO_2_ group, forming pi and negative ionizable interactions with the heme group, respectively. In the case of 5-adamantane thiadiazole-based thiazolidinones **[72]**, again, the most active compound form positive interactions between the heme group and heterocyclic rings of the compound. Thus, it can be concluded that thiazolidinone derivatives, in general, can interact with the heme of CYP51_Ca_ in the same way as ketoconazole interacts.

## 3. Materials and Methods

All starting materials were purchased from Merck and used without purification. NMR spectra were determined with Varian Mercury VX-400” (Varian Co., Palo Alto, CA, USA) and AM-300 Bruker 300 MHz. spectrometers in DMSO-d_6_. MS (ESI) spectra were recorded on an LC-MS system-HPLC Agilent 1100 (Agilent Technologies Inc., Santa, Clara, CA USA) equipped with a diode array detector Agilent LC\MSD SL. Parameters of analysis: Zorbax SB-C18 column (1.8 μM, 4.6–15 mm, PN 821975-932), solvent water–acetonitrile mixture (95:5), 0.1% of aqueous trifluoroacetic acid; eluent flow 3 mL min^–1^; injection volume 1 μL; IR spectra were recorded on a Vertex 70 Bruker” (Bruker, Karlsruhe, Germany) spectrometer in KBr pellets. Melting points were determined in open capillary tubes and are uncorrected.

### 3.1. In Silico Biological Activity Evaluation

Antimicrobial activity and toxicity of the designed compounds have been estimated in silico using web services available on the Way2Drug portal [56]. These services are based on the PASS (Prediction of Activity Spectra for Substances) and GUSAR (General Unrestricted Structure-Activity Relationships) software, which is described in detail elsewhere [60,61]. It is essential to mention that PASS-based services provide the assessments of the compound’s activity as the difference between the probabilities for the chemical compound with a particular structure to display activity (Pa) and do not display this activity (Pi). By default, in PASS, all activities with Pa > Pi are considered as probable. High Pa-Pi values reflect the high structural similarity of the analyzed compound to the structures included in the training set with those activities. Since our goal was not finding close analogs of the earlier discovered antimicrobial agents, we considered compounds with small Pa-Pi values as the promising hits for experimental testing. If the experiment will confirm their activity, there is a chance to find a New Chemical Entity. GUSAR-based service [60,61] provides the quantitative assessment of acute rat toxicity expressed as LD_50_ values for four routes of administration: intraperitoneal (IP), intravenous (IV), oral, and subcutaneous (SC).

### 3.2. Chemistry

#### 3.2.1. General Procedure for the Preparation of N-(4-oxo-2-thioxothiazolidin-3-yl) carbamides **3a**–**d**

In a round-bottom flask equipped with a reflux condenser, 0.05 mol of trithiocarbonyl diglycolic acid, 0.05 mol of the corresponding hydrazide and alcohol-water mixture (1:1) were placed and boiled for 3 h. The reaction mixture is cooled, the precipitate is filtered off and recrystallized.

**2-Hydroxy-N-(4-oxo-2-thioxothiazolidin-3-yl)benzamide 3a.** Yield 97%; m.p. 104–106 °C (CH_3_COOH-H_2_O 2:1). IR (cm^–1^): 3342.48 (OH), 1751.28 (C=O), 1657.74 (C=O), 1608.56 (C=S).). ^1^H NMR (400 MHz, DMSO-d_6,_ ppm) δ 11.62–10.91 (br.s, 2H, NH, OH), 7.88 (dd, J = 8.0, 1.6 Hz, 1H, H_6_ benzene), 7.52–7.46 (m, 1H, H_3_ benzene), 7.05–6.95 (m, 1H, 2H, H_4_ +H_5_, aromatic), 4.48 (q, J = 18.7 Hz, 2H, CH_2_). ^13^C NMR (101 MHz, DMSO, ppm) δ 199.90, 170.25, 164.91, 157.93, 134.62, 129.83, 119.44, 117.19, 115.07, 33.38. Anal. Calcd. for C_10_H_8_N_2_O_3_S_2_ (%): C, 44.77; H, 3.01; N, 10.44; S, 23.90 Found (%):C, 44.88; H, 3.09; N, 10.37; S, 23.95.

**4-Hydroxy-*N*-(4-oxo-2-thioxothiazolidin-3-yl)benzamide 3b.** Yield 87%; m.p. 207–209 °C (CH_3_COOH).). IR (cm^–1^): 3259.54 (OH), 3166(NH), 1739.71 (C=O), 1667.38(C=O), 1583.48 (C=S). ^1^H NMR (400 MHz, DMSO-d_6,_ ppm) δ 11.25 (s, 1H, NH), 10.29 (s, 1H, OH), 7.81 (dd, J = 9.1, 2.3 Hz, 2H, H_2_ +H_6_, benzene), 6.88 (dd, J = 9.1, 2.3 Hz, 2H, H_3_ +H_5_, benzene), 4.51 (s, 2H, CH_2_). ^13^C NMR (101 MHz, DMSO, ppm) δ 200.33, 170.54, 163.93, 161.44, 129.96, 121.44, 115.24, 33.32. Anal. Calcd. for C_10_H_8_N_2_O_3_S_2_ (%): C, 44.77; H, 3.01; N, 10.44; S, 23.90 Found (%):C, 44.69; H, 2.95; N, 10.36; S, 23.81.

**N-(4-Oxo-2-thioxothiazolidin-3-yl)nicotinamide 3c.** Yield 83%; m.p. 190 °C decomp.(C_2_H_5_OH). IR (cm^–1^): 1753.21 (C=O), 1687.63 (C=O), 1556.48 (C=S). ^1^H NMR (400 MHz, DMSO-d_6,_ ppm) δ 11.95 (s, 1H, NH), 8.97–8.73 (m, 2H, H_2_ +H_4_, pyridine), 7.99–7.70 (m, 2H, H_5_ +H_6_, pyridine), 4.55 (s, 2H, CH_2_).). ^13^C NMR (101 MHz, DMSO, ppm) δ 199.81, 170.20, 163.25, 150.76, 137.93, 121.31, 119.56, 33.55. Anal. Calcd. for C_9_H_7_N_3_O_2_S_2_ (%): C, 42.68; H, 2.79; N, 16.59; S, 25.32 Found (%):C, 42.79; H, 2.70; N, 16.48; S, 25.45.

**N-(4-Oxo-2-thioxothiazolidin-3-yl)isonicotinamide 3d**. Yield 85%; m.p. 193 °C decomp. (C_2_H_5_OH). IR (cm^–1^): 1753.21(C=O), 1678.95 (C=O), 1556.48 (C=S).^1^H NMR (400 MHz, DMSO-d_6,_ ppm) δ 11.95 (s, 1H, NH), 8.87–8.79 (m, 2H, H_3_ +H_5_, pyridine), 7.85–7.80 (m, 2H, H_2_ +H_6,_ pyridine), 4.55 (s, 2H, CH_2_). ^13^C NMR (101 MHz, DMSO, ppm) δ 199.81, 170.19, 163.25, 150.76, 137.93, 121.39, 119.56, 33.58. Anal. Calcd. for C_9_H_7_N_3_O_2_S_2_ (%): C, 42.68; H, 2.79; N, 16.59; S, 25.32 Found (%):C, 42.77; H, 2.85; N, 16.76; S, 25.26.

#### 3.2.2. General Procedure 5-[(R-1H-indol-3-ylmethylene)-4-oxo-2-thioxothiazolidin-3-yl] carbamides **5a–k** and 5-(R-1H-indol-3-ylmethylene)-3-morpholin-4-yl-2-thioxothiazolidin-4-ones **7a–c**

In a round-bottom flask equipped with a reflux condenser, 2.5 mmol of 3-substituted 2-thioxo-4-oxothiazolidine **3a-d** or **6**, 3.3 mmol of the corresponding aldehyde **1a-d,** 2.5 mmol of ammonium acetate and 5 mL of acetic acid are placed. The reaction mixture is boiled for 2 h, cooled, the precipitate is filtered off, washed with acetic acid and water, dried and recrystallized.

**2-Hydroxy-*N*-{(5*Z*)-5-[(1-methyl-1*H*-indol-3-yl)methylene]-4-oxo-2-thioxothiazolidin-3-yl}benzamide 5a.** Yield 98%; m.p. 265–267 °C (DMFA-CH_3_COOH). IR (cm^–1^): 3272.08 (OH), 1700.17 (C=O), 1656.77 (C=O), 1588.3 (C=C), 1573.84 (C=S). ^1^H-NMR (300 MHz, DMSO-d_6_, ppm) δ 11.40 (s, 1H, NH-CO), 11.31 (s, 1H, OH), 8.12 (s, 1H, CH=), 8.01 (d, *J* = 7.9 Hz, 1H, H_6_ benzene), 7.91 (d, *J* = 4.2 Hz, 2H, H_4_ +H_7_, indole), 7.54–7.41 (m, 2H, H_3_ benzene + H_2_ indole), 7.37–7.22 (m, 2H, H_5_ +H_6_, indole), 6.97 (dd, *J* = 17.3, 8.1 Hz, 2H, H_4_ +H_5_, benzene), 4.00 (s, 3H, CH_3_N). ^13^C NMR (101 MHz, DMSO, ppm) δ 189.71, 164.99, 163.08, 158.00, 136.96, 134.65, 134.45, 129.90, 127.29, 127.01, 123.54, 122.01, 119.46, 118.78, 117.22, 115.13, 111.01, 109.93, 33.50. ESI-MS [m/z]: [M + H]^+^ = 411.0; [M − H]^−^ = 408.2. Anal. Calcd. for C_20_H_15_N_3_O_3_S_2_ (%): C, 58.66; H, 3.69; N, 10.26; S, 15.66 Found (%): 58.75 H, 3.62; N, 10.21; S, 15.52.

**2-Hydroxy-*N*-{(5*Z*)-5-[(5-methoxy-1*H*-indol-3-yl)methylene]-4-oxo-2-thioxothiazolidin-3-yl}benzamide 5b.** Yield 77%; m.p. 239–241 °C (DMFA-CH_3_COOH).). IR (cm^–1^): 3234.47 (OH), 1699.21 (C=O), 1654.84 (C=O), 1585.41 (C=C, C=S). ^1^H-NMR (300 MHz, DMSO-d_6_, ppm) δ 12.15 (s, 1H, NH), 11.45 (s, 1H, NH-CO), 11.35 (s, 1H, OH), 8.18 (s, 1H, CH=), 8.00 (d, *J* = 7.8 Hz, 1H, H_6_ benzene), 7.74 (d, *J* = 2.2 Hz, 1H, H_4_ indole), 7.50–7.33 (m, 3H, H_3_ benzene + H_2_ +H_7_, indole), 7.04–6.90 (m, 2H, H_4_ +H_5_, benzene), 6.83 (d, *J* = 8.4 Hz, 1H, H_6_ indole), 3.87 (s, 3H, CH_3_O ^13^C NMR (101 MHz, DMSO, ppm) δ 189.72, 165.10, 163.07, 158.10, 155.45, 134.64, 131.12, 131.09, 129.80, 128.22, 127.84, 119.43, 117.25, 115.07, 113.67, 113.38, 111.09, 110.44, 100.58, 55.85. ESI-MS [m/z]: [M + H]^+^ = 426.0; [M − H]^−^ = 424.0. Anal. Calcd. for C_20_H_15_N_3_O_4_S_2_ (%): C, 56.46; H, 3.55; N, 9.88; S, 15.07 Found (%):C, 56.57; H, 3.49; N, 9.96; S, 15.15.

**2-Hydroxy-*N*-{(5*Z*)-5-[(6-methoxy-1*H*-indol-3-yl)methylene]-4-oxo-2-thioxothiazolidin-3-yl}benzamide 5c.** Yield 80%; m.p. 268–270 °C (DMFA-CH_3_COOH). IR (cm^–1^): 3227.72 (OH), 1705.96 (C=O), 1670.27 (C=O), 1591.2 (C=C), 1576.73 (C=S). ^1^H-NMR (300 MHz, DMSO-d_6_, ppm) δ 12.06 (s, 1H, NH), 11.45 (s, 1H, NH-CO),), 11.34 (s, 1H, OH), 8.10 (s, 1H, CH=), 7.99 (d, *J* = 7.9 Hz, 1H, H_6_ benzene), 7.75 (d, *J* = 8.7 Hz, 1H, H_4_ indole), 7.69 (d, *J* = 1.9 Hz, 1H, H_3_ benzene), 7.48-7,42 (m, 1H, H_2_ indole), 7.05–6.91 (m, 3H, H_7_ indole +H_4_ + H_5_, benzene), 6. 83 (d, *J* = 8.5 Hz, 1H, H_5_ indole), 3.84 (s, 3H, CH_3_O^13^C NMR (101 MHz, DMSO, ppm) δ 189.71, 165.00, 163.10, 158.01, 156.93, 137.32, 134.64, 130.31, 129.87, 127.93, 120.68, 119.49, 119.44, 117.22, 115.11, 111.63, 111.13, 111.06, 95.41, 55.28. ESI-MS [m/z]: [M + H]^+^ = 426.0; [M − H]^−^ = 424.0.. Anal. Calcd. for C_20_H_15_N_3_O_4_S_2_ (%): C, 56.46; H, 3.55; N, 9.88; S, 15.07 Found (%):C, 56.39; H, 3.51; N, 9.80; S, 15.01.

**4-Hydroxy-*N*-[(5*Z*)-5-(1*H*-indol-3-ylmethylene)-4-oxo-2-thioxothiazolidin-3-yl]benzamide 5d.** Yield 90%; m.p. > 275 °C (DMFA-CH_3_COOH). IR (cm^–1^): 3369.48 (OH), 3225.79 (NH), 1694.38 (C=O), 1668.35 (C=O), 1591.2 (C=C), 1573.84 (C=S). ^1^H-NMR (300 MHz, DMSO-d_6_, ppm) δ 12.23 (s, 1H, NH), 11.12 (s, 1H, NH-CO), 9.89 (s, 1H, OH), 8.13 (s, 1H, CH=), 7.92–7.81 (m, 3H, H_2_ + H_6_, benzene + H_4_ indole), 7.79 (d, *J* = 3.0 Hz, 1H, H_7_ indole), 7.53–7.45 (m, 1H, H_2_ indole), 7.28–7.15 (m, 2H, H_5_ + H_6_, indole), 6.85 (d, *J* = 8.7 Hz, 2H, H_3_ +H_5_, benzene). ^13^C NMR (101 MHz, DMSO, ppm) δ 190.12, 164.06, 163.36, 161.49, 136.48, 131.20, 129.97, 127.80, 126.77, 123.49, 121.69, 121.50, 118.64, 115.30, 112.61, 111.16, 110.97. ESI-MS [m/z]: [M + H]^+^ = 396.0; [M − H]^−^ = 394.0. Anal. Calcd. for C_19_H_13_N_3_O_3_S_2_ (%): C, 57.71; H, 3.31; N, 10.63; S, 16.22 Found (%):C, 57.62; H, 3.37; N, 10.55; S, 16.16.

***N*-[(5*Z*)-5-(1*H*-Indol-3-ylmethylene)-4-oxo-2-thioxothiazolidin-3-yl]nicotinamide 5e.** Yield 90%; m.p. > 275 °C (DMFA-CH_3_COOH). IR (cm^–1^): 3485.2 (NH), 1696.31 (C=O), 1674.13 (C=O), 1596.98 (C=C), 1577.7 (C=S). ^1^H-NMR (500 MHz, DMSO-d_6_, ppm) δ 12.31 (s, 1H, NH), 11.75 (s, 1H, NH-CO), 9.15 (d, *J* = 1.7 Hz, 1H, H_4_ pyridine), 8.77 (dd, *J* = 4.8, 1.4 Hz, 1H, H_2_ pyridine), 8.33 (d, *J* = 8.0 Hz, 1H, H_6_ pyridine), 8.17 (s, 1H, CH=), 7.90 (d, *J* = 7.6 Hz, 1H, H_5_ pyridine), 7.84 (d, *J* = 3.0 Hz, 1H, H_4_ indole), 7.57–7.48 (m, 2H, H_2_ +H_7_, indole), 7.27–7.18 (m, 2H, H_5_ +H_6_, indole). ^13^C NMR (101 MHz, DMSO, ppm) δ 189.54, 163.32, 162.94, 150.78, 137.89, 136.41, 131.49, 128.38, 126.78, 123.56, 121.78, 121.38, 118.68, 112.65, 110.98, 110.72. ESI-MS [m/z]: [M + H]^+^ = 381.0; Anal. Calcd. for C_18_H_12_N_4_O_2_S_2_ (%): C, 56.83; H, 3.18; N, 14.73; S, 16.86 Found (%):C, 56.71; H, 3.24; N, 14.80; S, 16.79.

***N*-{(5*Z*)-5-[(1-Methyl-1*H*-indol-3-yl)methylene]-4-oxo-2-thioxothiazolidin-3-yl}nicotinamide 5f.** Yield 94%; m.p. 270–272 °C (DMFA-CH_3_COOH). IR (cm^–1^): 3241.22 (NH), 1706.92 (C=O), 1681.85 (C=O), 1589.27 (C=C), 1572.87 (C=S). ^1^H-NMR (300 MHz, DMSO-d_6_, ppm) δ 11.75 (s, 1H, NH-CO), 9.16 (d, *J* = 1.9 Hz, 1H, _H_4__ pyridine), 8.77 (dd, *J* = 4.8, 1.3 Hz, 1H, H_2_ pyridine), 8.36–8.31 (m, 1H, H_6_ pyridine), 8.12 (s, 1H, CH=), 7.96 (s, 1H, H_5_ pyridine), 7.91 (d, 1H, *J* = 7.9 Hz, H_4_ indole), 7.58–7.47 (m, 2H, H_2_ + H_7_, indole), 7.32 (t, *J* = 7.5 Hz, 1H, H_6_ indole), 7.26 (t, *J* = 7.4 Hz, 1H, H_5_ indole), 4.00 (s, 3H, CH_3_N). ^13^C NMR (101 MHz, DMSO, ppm) δ 189.64, 163.34, 163.01, 153.40, 148.60, 136.99, 135.59, 134.68, 127.57, 127.30, 126.77, 123.97, 123.60, 122.10, 118.80, 111.08, 110.55, 109.95, 33.54. ESI-MS [m/z]: [M + H]^+^ = 395.0; [M − H]^−^ = 394.0. Anal. Calcd. for C_19_H_14_N_4_O_2_S_2_ (%): C, 57.85; H, 3.58; N, 14.20; S, 16.26 Found (%):C, 57.94; H, 3.51; N, 14.15; S, 16.35.

***N*-{(5*Z*)-5-[(5-Methoxy-1*H*-indol-3-yl)methylene]-4-oxo-2-thioxothiazolidin-3-yl}nicotinamide 5g.** Yield 00%; m.p. 199–201 °C. IR (cm^–1^): 3254.72 (NH), 1718.49 (C=O), 1681.85 (C=O), 1585.41 (C=C), 1576.73 (C=S). ^1^H-NMR (300 MHz, DMSO-d_6_, ppm) ^1^H-NMR (300 MHz, DMSO-d_6_, ppm) δ 12.17 (s, 1H, NH), 11.72 (s, 1H, NH-CO), 9.17 (s, 1H, H_4_ pyridine), 8.77 (d, *J* = 3.0 Hz, 1H, H_2_ pyridine), 8.35 (d, *J* = 7.5 Hz, 1H, H_6_ pyridine), 8.19 (s, 1H, CH=), 7.74 (s, 1H, H_5_ pyridine), 7.59–7.49 (m, 1H, H_2_ indole), 7.46–7.30 (m, 2H, H_4_ +H_7_ indole), 6.83 (d, *J* = 8.5 Hz, 1H, H_6_ indole), 3.86 (s, 3H, CH_3_O). ^13^C NMR (101 MHz, DMSO, ppm) δ 189.67, 163.30, 163.01, 155.51, 153.36, 148.61, 135.57, 131.33, 131.15, 128.76, 127.86, 126.82, 123.94, 113.69, 113.41, 111.12, 110.01, 100.64, 55.54. ESI-MS [m/z]: [M + H]^+^ = 411.0; [M − H]^−^ = 409.0. Anal. Calcd. for C_19_H_14_N_4_O_3_S_2_ (%): C, 55.60; H, 3.44; N, 13.65; S, 15.62 Found (%): C, 55.49; H, 3.39; N, 13.58; S, 15.67.

***N*-[(5*Z*)-5-(1*H*-Indol-3-ylmethylene)-4-oxo-2-thioxothiazolidin-3-yl]isonicotinamide 5h.** Yield 86%; m.p. > 275 ºC Yield 86%; m.p. > 275 °C (DMFA-CH_3_COOH). IR (cm^–1^): 3196.86 (NH), 1718.49 (C=O), 1672.2 (C=O), 1594.09 (C=C), 1576.73 (C=S). ^1^H-NMR (300 MHz, DMSO-d_6_, ppm) δ 12.27 (s, 1H, NH), 11.79 (s, 1H, NH-CO), 8.78 (d, *J* = 5.8 Hz, 2H, H_2_ +H_6_, pyridine), 8.17 (s, 1H, CH=), 7.94–7.86 (m, 3H, H_3_ +H_5_, pyridine +H_4_ indole), 7.82 (s, 1H, H_7_ indole), 7.51 (d, *J* = 7.1 Hz, 1H, H_2_ indole), 7.30–7.15 (m, 2H, H_5_ +H_6_, indole). ^13^C NMR (101 MHz, DMSO, ppm) δ 189.53, 163.32, 162.94, 150.76, 137.94, 136.42, 131.46, 128.34, 126.77, 123.54, 121.76, 121.38, 118.66, 112.65, 110.99, 110.78. ESI-MS [m/z]: [M + H]^+^ = 381.0; [M − H]^−^ = 379.0. Anal. Calcd. for C_18_H_12_N_4_O_2_S_2_ (%): C, 56.83; H, 3.18; N, 14.73; S, 16.86 Found (%):C, 56.89; H, 3.26; N, 14.65; S, 16.88.

***N*-{(5*Z*)-5-[(1-Methyl-1*H*-indol-3-yl)methylene]-4-oxo-2-thioxothiazolidin-3-yl}isonicotinamide 5i.** Yield 95%; m.p. 269–271 °C (DMFA-CH_3_COOH). IR (cm^–1^): 3217.11 (NH), 1710.78 (C=O), 1674.13 (C=O), 1587.34 (C=C), 1570.95 (C=S).^1^H-NMR (300 MHz, DMSO-d_6_, ppm) δ 11.81 (s, 1H, NH-CO), 8.79 (d, *J* = 5.9 Hz, 2H, H_2_ +H_6_, pyridine), 8.13 (s, 1H, CH=), 7.99–7.86 (m, 4H, H_3_ +H_5_, pyridine + H_4_ +H_7_, indole), 7.51 (d, *J* = 7.9 Hz, 1H, H_2_ indole), 7.37–7.20 (m, 2H, H_5_ +H_6_, indole), 4.00 (s, 3H, CH_3_N). ^13^C NMR (101 MHz, DMSO, ppm) δ 189.49, 163.32, 162.90, 150.78, 137.88, 137.00, 134.73, 127.68, 127.30, 123.61, 122.12, 121.38, 118.81, 111.09, 110.46, 109.95, 33.55. ESI-MS [m/z]: [M + H]^+^ = 395.0; [M − H]^−^ = 393.0. Anal. Calcd. for C_19_H_14_N_4_O_2_S_2_ (%): C, 57.85; H, 3.58; N, 14.20; S, 16.26 Found (%):C, 57.78; H, 3.53; N, 14.28; S, 16.17.

***N*-{(5*Z*)-5-[(5-Methoxy-1*H*-indol-3-yl)methylene]-4-oxo-2-thioxothiazolidin-3-yl}isonicotinamide 5j.** Yield 89%; m.p. 261–263 ºC (CH_3_COOH).). IR (cm^–1^): 3199.75 (NH), 1706.92 (C=O), 1676.06 (C=O), 1588.3 (C=C, C=S). ^1^H-NMR (300 MHz, DMSO-d_6_, ppm) δ 12.20 (s, 1H, NH), 11.83 (s, 1H, NH-CO), 8.78 (d, *J* = 5.4 Hz, 2H, H_2_ +H_6_, pyridine), 8.20 (s, 1H, CH=), 7.90 (d, *J* = 5.4 Hz, 2H, H_3_ +H_5_, pyridine), 7.76 (d, *J* = 3.0 Hz, 1H, H_4_ indole), 7.44–7.33 (m, 2H, H_2_ +H_7_, indole), 6.83 (dd, *J* = 8.9, 1.7 Hz, 1H, H_6_ indole), 3.86 (s, 3H, CH_3_O). ^13^C NMR (101 MHz, DMSO, ppm) δ 189.50, 163.29, 162.91, 155.49, 150.78, 137.91, 131.39, 131.12, 128.89, 127.88, 121.39, 113.72, 113.42, 111.12, 109.87, 100.58, 55.51. ESI-MS [m/z]: [M + H]^+^ = 411.0; [M − H]^−^ = 409.0. Anal. Calcd. for C_19_H_14_N_4_O_3_S_2_ (%): C, 55.60; H, 3.44; N, 13.65; S, 15.62 Found (%): C, 55.52; H, 3.47; N, 13.73; S, 15.55.

***N*-{(5*Z*)-5-[(6-Methoxy-1*H*-indol-3-yl)methylene]-4-oxo-2-thioxothiazolidin-3-yl}isonicotinamide 5k.** Yield 89%; m.p. 275–277 °C (CH_3_COOH). IR (cm^–1^): 3550.78 (NH), 3346.34 (NH), 1725.24 (C=O), 1689.56 (C=O), 1596.98 (C=C), 1576.73 (C=S). ^1^H-NMR (300 MHz, DMSO-d_6_, ppm) δ 12.11 (s, 1H, NH), 11.85 (s, 1H, NH-CO), 8.78 (d, *J* = 5.3 Hz, 2H,H_2_ +H_6_, pyridine), 8.11 (s, 1H, CH=), 7.89 (d, *J* = 5.3 Hz, 2H, H_3_ +H_5_, pyridine), 7.74 (dd, *J* = 13.7, 5.6 Hz, 2H, H_2_ +H_4_, indole), 6.96 (s, 1H, H_7_ indole), 6.83 (d, *J* = 8.6 Hz, 1H, H_5_ indole), 3.84 (s, 3H, CH_3_O). ^13^C NMR (101 MHz, DMSO, ppm) δ 189.49, 163.30, 162.91, 156.97, 150.78, 137.88, 137.36, 130.66, 128.61, 121.38, 120.66, 119.52, 111.71, 111.17, 110.52, 95.46, 55.29. ESI-MS [m/z]: [M + H]^+^ = 411.0; [M − H]^−^ = 409.0. Anal. Calcd. for C_19_H_14_N_4_O_3_S_2_ (%): C, 55.60; H, 3.44; N, 13.65; S, 15.62 Found (%): C, 55.73; H, 3.49; N, 13.57; S, 15.54.

**(5*Z*)-5-(1*H*-Indol-3-ylmethylene)-3-morpholin-4-yl-2-thioxothiazolidin-4-one7a.** Yield 86%; m.p. 273–275 °C (DMFA:CH_3_COOH). IR (cm^–1^): 3247.97 (NH), 1690.53 (C=O), 1594.09 (C=C), 1575.77 (C=S).^1^H-NMR (300 MHz, DMSO-d_6_, ppm) δ 12.10 (s, 1H, NH), 7.98 (s, 1H, CH=), 7.85 (d, *J* = 6.8 Hz, 1H, H_4_ indole), 7.66 (d, *J* = 2.8 Hz, 1H, H_7_ indole), 7.51–7.45 (m, 1H, H_2_ indole), 7.27–7.13 (m, 2H, H_5_ +H_6_, indole), 3.81 (s, 6H, morpholine), 3.06 (s, 2H, morpholine). ^13^C NMR (101 MHz, DMSO, ppm) δ 189.92, 165.21, 136.32, 130.51, 126.72, 126.03, 123.34, 121.50, 118.48, 112.53, 111.68, 110.95, 66.56, 50.14. ESI-MS [m/z]: [M + H]^+^ = 346.2; [M − H]^−^ = 344.2. Anal. Calcd. for C_16_H_15_N_3_O_2_S_2_ (%): C, 55.63; H, 4.38; N, 12.16; S, 18.56 Found (%):C, 55.74; H, 4.32; N, 12.24; S, 18.49.

(5*Z*)-5-[(1-Methyl-1*H*-indol-3-yl)methylene]-3-morpholin-4-yl-2-thioxo-thiazolidin-4-one 7b was prepared according to [42].

**(5*Z*)-5-[(5-Methoxy-1*H*-indol-3-yl)methylene]-3-morpholin-4-yl-2-thioxo-thiazolidin-4-one7c.** Yield 82%; m.p. 250–252 °C (CH_3_COOH). IR (cm^–1^): 3163.11 (NH), 1690.53 (C=O), 1580.59 (C=C, C=S). ^1^H-NMR (300 MHz, DMSO-d_6_, ppm) δ 12.08 (s, 1H, NH), 7.99 (s, 1H, CH=), 7.61 (s, 1H, H_4_ indole), 7.34 (d, *J* = 3.4 Hz, 2H, H_2_ +H_7_, indole), 6.81 (d, *J* = 8.7 Hz, 1H, H_6_ indole), 4.05–3.57 (m, 9H, CH_3_O, morpholine), 3.03 (s, 2H, morpholine). ^13^C NMR (101 MHz, DMSO, ppm) δ 189.87, 165.20, 155.29, 131.04, 130.51, 127.75, 126.61, 113.52, 113.30, 111.04, 110.74, 100.34, 66.56, 55.46, 50.11. ESI-MS [m/z]: [M + H]^+^ = 376.0; [M − H]^−^ = 374.0. Anal. Calcd. for C_17_H_17_N_3_O_3_S_2_ (%): C, 54.38; H, 4.56; N, 11.19; S, 17.08 Found (%):C, 54.31; H, 4.62; N, 11.04; S, 17.15.

### 3.3. Antibacterial Activity Evaluation

Bacterial strains utilized include Gram-negative: *Salmonella typhimurium* (ATCC 13311) *Pseudomonas aeruginosa* (ATCC 27853), *Escherichia coli* (ATCC 35210), *Enterobacter cloacae* (ATCC 35030) and Gram-positive bacteria: *Micrococcus flavus* (ATCC 10240), *Bacillus cereus* (isolated clinically), *Staphylococcus aureus* (ATCC 6538), and *Listeria monocytogenes* (NCTC 7973) bacteria. Pathogens were provided from the Mycological Laboratory, Institute for Biological Research “Siniša Stankovic” National institute of Republic of Serbia Belgrade. Resistant strains used were MRSA, *E. coli*, and *P. aeruginosa* [77,78].

For the determination of minimum inhibitory (MIC) and minimum bactericidal concentrations, the microdilution method, as previously described [77,78,79]. The minimum inhibitory (MIC) and minimum bactericidal (MBC) concentrations were determined by the modified microdilution method as previously reported [77,78,79]. Briefly, the fresh overnight culture of bacteria was adjusted to a concentration of 1 × 10^5^ CFU/mL. The tested compounds were dissolved in 5% DMSO and serially diluted in tryptic soy broth (TSB) medium with bacterial inoculum (1.0 × 10^4^ CFU per well). The microplates were incubated for 24 h at 37 °C. The MIC of the samples was detected following the addition of 40 μL of iodonitrotetrazolium chloride (INT) (0.2 mg/mL) and incubation at 37 °C for 30 min. The lowest concentration that produced a significant inhibition of the growth of the bacteria in comparison with the positive control was identified as the MIC. MBC was determined by serial sub-cultivation of 10 μL into microplates containing 100 μL of TSB. The lowest concentration that shows no growth after this sub-culturing was identified as the MBC, indicating 99.5% death of the original inoculum. Streptomycin and ampicillin were used as positive controls.

### 3.4. Antifungal Evaluation

The following fungi were used: Aspergillus niger (ATCC 6275), Aspergillus ochraceus (ATCC 12066), Aspergillus fumigatus (human isolate), Aspergillus versicolor (ATCC 11730), Penicillium funiculosum (ATCC 36839), Penicillium ochrochloron (ATCC 9112), Trichoderma viride (IAM 5061), Penicillium verrucosum var. cyclopium (food isolate). The organisms were obtained from the Mycological Laboratory, Department of Plant Physiology, Institute for Biological Research “Siniša Stankovic”, National institute of Republic of Serbia, Belgrade, Serbia. All experiments were performed in duplicate and repeated three times, as previously described [80,81].

The fungal spores were washed from the surface of agar plates with sterile 0.85% saline containing 0.1% Tween 80 (*v/v*). The spore suspension was adjusted with sterile saline to a concentration of approximately 1.0 × 10^5^ in a final volume of 100 μL per well. MIC determinations were performed by a serial dilution technique using 96-well microtiter plates. The examined compounds were serially diluted in broth Malt medium (MA), after which inoculum was added. The microplates were incubated for 72 h at 28 °C. The lowest concentrations without visible growth (at the binocular microscope) were defined as MICs. The fungicidal concentrations (MFCs) were determined by serial subcultivation of 2 μL of tested fractions dissolved in medium and inoculation into microtiter plates containing 100 μL of broth per well and further incubation 72 h at 28 °C. The lowest concentration with no visible growth was defined as MFC, indicating 99.5% killing of the original inoculum. The fungicides bifonazole and ketoconazole were used as positive controls.

### 3.5. Docking Studies

Τhe program AutoDock 4.2^®^ software was used for the docking simulation. The free energy of binding (ΔG) of *E. coli* DNA GyrB, Thymidylate kinase, *E. coli* MurA, *E. coli* primase, *E. coli* MurB, DNA topo IV, and CYP51 of *C*. *albicans,* in complex with the inhibitors were generated using this molecular docking program. The X-ray crystal structures data of all the enzymes used were obtained from the Protein Data Bank (PDB ID: 1KZN, AQGG, 1DDE, JV4T, 2Q85, 1S16, and 5V5Z respectively). All procedures were performed according to our previous paper [78].

### 3.6. Cytotoxicity

**HEK 293** cells were cultured in DMEM medium, supplemented with 10% fetal calf serum (Sigma Chemical Co., St. Louis, MO, USA), 50 µg/mL streptomycin (Sigma Chemical Co.), and 50 units/mL penicillin (Sigma Chemical Co.) in 5% CO_2_-containing humidified atmosphere at 37°C. Since compound solutions contained DMSO, control cultures containing only DMSO at the final concentration obtained when the appropriate volume of compound solution was added were performed.

#### MTT Assay for Determination of Cell Viability

MTT assay based on the colorimetric measurement of formazan formed after reducing MTT by cellular NAD(P)H-dependent oxidoreductases was used to examine the cytotoxic activity of the compounds. Briefly, the cells were seeded into 96-well plates in 100 μL of complete culture medium at a concentration of 5,000 substrate-dependent cells per well and left incubated overnight as described above. The formulations to be tested (100 μL aliquots) were added to the culture medium at different concentrations and left incubated for 72 h. The MTT assay was performed following the manufacturer’s recommendations and assessed using an EL ×800 absorbance reader (BioTek Instruments; Winooski, VT, USA).

## 4. Conclusions

Eleven 5-[(R-1*H*-indol-3-yl)methylene]-4-oxo-2-thioxo-thiazolidin-3-ylcarbamides **5a-k** and three 5-[(R-1*H*-indol-3-yl) methylene] -3-morpholin-4-yl-2-thioxothiazolidin-4-ones **7a-c** were designed, synthesized and evaluated in silico and experimentally for their antimicrobial action against panel of Gram positive, Gram negative bacteria and fungi.

It should be mentioned that all compounds appeared to be more potent than ampicillin against all bacteria tested and then streptomycin against all bacteria except *B. cereus (isolated clinically M. flavus* (ATCC 10240)*,* and *En. cloacae* (ATCC 35030). The most sensitive bacteria was found to be *S. aureus* (ATCC 6538), while *L. monocytogenes* (NCTC 7973) was the most resistant one. Compounds also appeared to be active against three resistant strains MRSA, *E. coli,* and *P. aeruginosa* showing better activity against MRSA than both reference drugs while against the other two resistant strains better than ampicillin.

Concerning antifungal action, the tested compounds exhibited very good activity against all the fungal species tested, being more active than ketoconazole and bifonazole. The most sensitive fungal strain appeared to be *T. viride* (IAM 5061), while the most resistant filamentous *A. fumigatus* (human isolate).

It can be observed that the growth of both Gram-negative and Gram-positive bacteria and fungi responded differently to the tested compounds, which indicates that different substituents may lead to different modes of action or that the metabolism of some bacteria/fungi was better able to overcome the effect of the compounds or adapt to it.

Docking analysis to DNA Gyrase, Thymidylate kinase and *E.coli* MurB indicated a probable involvement of MurB inhibition in the antibacterial mechanism of compounds tested while docking analysis to 14α-lanosterol demethylase (CYP51) and tetrahydrofolate reductase of *Candida albicans* indicated a likely implication of CYP51 reductase at the antifungal activity of the compounds and secondary involvement of dihydrofolate reductase inhibition at the mechanism of action of the most active compounds.

Since the most active compounds **5d**, **5g**, **5k**, **7c** demonstrated the low cytotoxicity against HEK-293 human embryonic kidney cell line and reasonable selectivity index, this chemical series looks promising for investigations as the antimicrobial agents.

Finally, compounds **5d** (Z)-N-(5-((1H-indol-3-yl)methylene)-4-oxo-2-thioxothiazolidin-3 -yl)-4-hydroxybenzamide and **5g** (Z)-N-(5-((5-methoxy-1H-indol-3-yl)methylene)-4-oxo-2-thioxothiazolidin-3-yl)nicotinamide as well as **7c** (Z)-5-((5-methoxy-1H-indol-3-yl)methylene)-3-morpholino-2-thioxothiazolidin-4-one can be considered as lead compounds for further development of more potent and safe antibacterial and antifungal agents.

## Figures and Tables

**Table 1:** In silico assessments of acute rat toxicity.

Compound ID	LD_50_, mg/kg				Toxicity Class			
	IP	IV	Oral	SC	IP	IV	Oral	SC
**5a**	809.8	402.5	1218	780.4 *	5	5	4	4 *
**5b**	680.4	309	1266	1434	5	5	4	5
**5c**	980.3	311.5	1325	619.9 *	5	5	4	4 *
**5d**								
**5e**	1263	466	843.9	440 *	NT	5	4	4 *
**5f**	1266	502.2	469.2	477.7 *	NT	5	4	4 *
**5g**	1010 *	371.9	192.4 *	397.6 *	5 *	5	3 *	4 *
**5h**	1282	448.5	1001	1588	NT	5	4	5
**5i**	1299	476.5	732.3	545.4 *	NT	5	4	4 *
**5j**	1258	381.6	196.2 *	422 *	NT	5	3 *	4 *
**5k**	1031 *	398.3	202.8 *	442 *	5 *	5	3 *	4 *
**7a**	1033	236.6	593.7	1644 *	5	4	4	5 *
**7b**	1061	287.2	720 *	862.1 *	5	4	4 *	4 *
**7c**	1180 *	210.7	765.1 *	682 *	5 *	4	4 *	4 *

**Notes:** *: Calculated for compounds that do not correspond to the model’s applicability domain; thus, they are less reliable than unmarked ones. NT: Non-Toxic.

**Table 2:** Antibacterial activity of compounds **5a–k** and **7a–c** (MIC/MBC in μM).

Com/d ID		*B.c*	*M.f*	*S.a*	*L.m*	*En.cl*	*P.a*	*S.T*	*E.coli*
**5a**	**MIC**	73.3 ± 0.4	109.9 ± 0.1	73.3 ± 0.3	146.5 ± 1.0	73.3 ± 0.8	73.3 ± 0.08	73.3 ± 0.08	109.9 ± 0.1
	**MBC**	146.5 ± 1.0	146.5 ± 2.0	146.5 ± 2.0	293.0 ± 4.0	146.5 ± 1.0	146.5 ± 1.0	146.5 ± 1.0	146.5 ± 2.0
**5b**	**MIC**	70.5 ± 0.4	105.8 ± 0.8	70.5 ± 0.8	105.8 ± 1.5	70.5 ± 0.8	70.5 ± 0.8	141.0 ± 1.2	211.5 ± 2.0
	**MBC**	141.0 ± 1.0	141.0 ± 2.0	141.0 ± 1.0	141.0 ± 2.0	141.0 ± 1.0	141.0 ± 0.1	282.0 ± 3.0	282.0 ± 2.0
**5c**	**MIC**	68.2 ± 0.8	102.6 ± 1.0	68.4 ± 0.5	68.4 ± 0.4	68.4 ± 0.4	68.4 ± 0.4	102.6 ± 1.0	102.6 ± 1.5
	**MBC**	136.8 ± 1.0	136.8 ± 1.5	136.8 ± .1.0	136.8 ± 1.0	136.8 ± 1.0	136.8 ± 1.0	136.8 ± 1.5	136.8 ± 2.0
**5d**	**MIC**	55.6 ± 0.2	113.8 ± 0.8	37.9 ± 0.2	113.8 ± 0.8	37.9 ± 0.4	37.9 ± 0.2	113.8 ± 1.0	75.6 ± 0.4
	**MBC**	75.9 ± 0.4	151.7 ± 2.0	75.9 ± 0.5	151.7 ± 2.0	75.9 ± 0.8	75.9 ± 0.6	151.7 ± 1.0	151.7 ± 1.0
**5e**	**MIC**	78.9 ± 0.2	118.3 ± 1.0	57.8 ± 0.4	78.9 ± 0.5	78.9 ± 0.1	78.9 ± 0.6	78.9 ± 0.8	118.3 ± 1.0
	**MBC**	157.7 ± 0.8	157.7 ± 2.0	78.9 ± 0.8	157.7 ± 1.5	157.7 ± 2.0	157.7 ± 1.5	157.7 ± 1.0	157.7 ± 2.0
**5f**	**MIC**	114.1 ± 1.0	114.1 ± 8.0	114.1 ± 1.0	76.1 ± 0.4	76.1 ± 0.8	76.1 ± 0.3	114.1 ± 1.5	114.1 ± 1.0
	**MBC**	152.1 ± 2.0	152.1 ± 1.0	152.1 ± 1.5	152.1 ± 1.0	152.1 ± 1.0	152.1 ± 1.0	152.1 ± 2.0	152.1 ± 1.0
**5g**	**MIC**	73.1 ± 1.0	73.1 ± 1.0	53.6 ± 0.4	73.1 ± 0.8	36.5 ± 0.5	109.6 ± 1.0	53.6 ± 0.6	109.6 ± 1.0
	**MBC**	146.2 ± 1.0	146.2 ± 1.0	73.1 ± 0.8	146.2 ± 1.6	73.1 ± 1.0	146.2 ± 1.2	73.1 ± 1.0	146.2 ± 2.0
**5h**	**MIC**	78.9 ± 0.5	118.3 ± 1.5	118.3 ± 1.0	78.9 ± 0.8	39.4 ± 0.5	39.4 ± 0.6	78.9 ± 0.6	118.3 ± 1.5
	**MBC**	157.7 ± 1.0	157.7 ± 2.0	157.7 ± 2.0	157.7 ± 1.0	78.9 ± 0.8	78.9 ± 0.8	157.7 ± 1.2	157.7 ± 1.0
**5i**	**MIC**	114.1 ± 1.0	114.1 ± 1.5	76.1 ± 0.8	152.1 ± 1.0	76.1 ± 0.8	76.1 ± 0.8	152.1 ± 1.0	114.1 ± 1.0
	**MBC**	152.1 ± 1.0	152.1 ± 2.0	152.1 ± 2.0	304.2 ± 4.0	152.1 ± 1.2	152.1 ± 1.2	304.2 ± 2.0	152.1 ± 1.0
**5j**	**MIC**	73.1 ± 0.5	109.6 ± 1.0	58.6 ± 0.4	146.2 ± 0.8	58.6 ± 0.6	58.6 ± 0.8	109.6 ± 1.0	109.6 ± 1.0
	**MBC**	146.2 ± 1.0	146.2 ± 2.0	73.1 ± 0.8	292.3 ± 0.2	73.1 ± 0.6	73.1 ± 0.06	146.2 ± 1.0	146.2 ± 2.0
**5k**	**MIC**	73.1 ± 0.5	109.6 ± 1.0	58.6 ± 0.4	109.6 ± 1.5	58.6 ± 0.6	58.6 ± 0.6	109.6 ± 1.5	109.6 ± 2.0
	**MBC**	146.2 ± 1.0	146.2 ± 2.0	73.1 ± 0.8	146.2 ± 1.0	73.1 ± 0.8	73.1 ± 0.8	146.2 ± 2.0	146.2 ± 2.0
**7a**	**MIC**	130.3 ± 1.0	130.3 ± 1.5	63.7 ± 0.4	86.9 ± 0.4	86.9 ± 1.0	86.9 ± 1.0	173.7 ± 2.0	130.3 ± 2.0
	**MBC**	173.7 ± 2.0	173.7 ± 2.0	86.9 ± 0.8	173.7 ± 1.5	173.7 ± 1.5	173.7 ± 1.5	347.4 ± 4.0	173.7 ± 1.5
**7b**	**MIC**	41.7 ± 0.2	125.2 ± 1.0	41.7 ± 0.2	166.9 ± 1.0	83.4 ± 0.9	61.2 ± 0.5	166.9 ± 2.0	125.2 ± .1.0
	**MBC**	83.4 ± 0.4	166.9 ± 2.0	83.4 ± 0.8	333.9 ± .2.0	166.9 ± 1.0	83.4 ± 1.0	333.9 ± 4.0	166.9 ± 2.0
**7c**	**MIC**	79.9 ± 0.4	119.8 ± 1.5	79.9 ± 1.0	159.8 ± 1.0	58.6 ± 0.4	58.6 ± 0.4	119.8 ± 1.0	119.8 ± 1.5
	**MBC**	159.8 ± 1.0	159.8 ± 2.0	159.8 ± 1.4	319.6 ± 2.0	79.9 ± 1.0	79.9 ± 1.0	159.8 ± 2.0	159.8 ± .2.0
**Am.**	**MIC**	248.0 ± 3.0	248.0 ± 2.0	248.0 ± 2.0	372.0 ± 4.0	248.0 ± 3.0	744.0 ± 9.0	248.0 ± 3.0	372.0 ± 4.0
	**MBC**	372.0 ± 4.0	372.0 ± 4.0	372.0 ± 2.0	744.0 ± 8.0	372.0 ± 3.0	1240 ± 2	492.0 ± 6.0	492.0 ± 8.0
**Str.**	**MIC**	43.0 ± 0.8	86.0 ± 1.0	172.0 ± 2.0	258.0 ± 4.0	43.0 ± 0.3	172.0 ± 3.0	172.0 ± 3.0	172.0 ± 2.0
	**MBC**	86.0 ± 1.0	172.0 ± 2.0	344.0 ± 4.0	516.0 ± 4.0	86.0 ± 0.6	344.0 ± 3.0	344.0 ± 3.0	344.0 ± 2.0

MIC–minimal inhibitory concentration, MBC–minimal bactericidal concentration, *B.c.-B.cereus* (clinical isolate), *M.f.-M.flavus* (ATCC 10,240), *S.a.-S.aurues* (ATCC 6538), *l.m.-L.monocytogenes* (NCTC 7973), *E.c.-E.coli* (ATTC 35210, *En.c.-En.cloaca* (ATCC 3503), *P.a.-P.aeruginosa* (ATCC 27,853), *S.T.-S.Typhimurium* (ATCC 13,311).

**Table 3:** Antibacterial activity against resistant strains (MIC/MBC in μM)**.**

Compound ID		Resistant Strains		
		MRSA	*P.aeruginosa*	*E.coli*
**5d**	**MIC**	1260 ± 0.8	315 ± 9.0	1260 ± 21
	**MBC**	2520 ± 0.1	630 ± 8.0	2502 ± 22
**5g**	**MIC**	1220 ± 18	610 ± 5.0	1220 ± 19
	**MBC**	2440 ± 0.2	1202 ± 21	2440 ± 16
**5k**	**MIC**	1220 ± 0.6	610 ± 10	1220 ± 0.6
	**MBC**	2440 ± 22	1220 ± 21	2440 ± 22
**Streptomycin**	**MIC**	172.0 ± 21	86 ± 12	172 ± 21
	**MBC**	-	172 ± 14	344 ± 42
**Ampicilline**	**MIC**	-	572 ± 64	572 ± 78
	**MBC**	/	/	/

**Table 4:** Antifungal activity of compounds **5a–k** and **7a–c** (MIC/MFC in μM).

Com. ID		*A.f*	*A.v*	*A.o*	*A.n*	*T.v*	*P.o*	*P.f*	*Pvc*
**5a**	**MIC**	293.0 ± 2.2	36.6 ± 0.4	26.9 ± 0.1	53.7 ± 0.6	26.9 ± 0.1	36.6 ± 0.2	36.6 ± 0.2	109.9 ± 0.1
	**MFC**	586.1 ± 7.0	73.3 ± 0.8	36.6 ± 0.2	73.3 ± 0.8	36.6 ± 0.3	73.3 ± 0.5	73.3 ± 0.8	146.5 ± 0.2
**5b**	**MIC**	35.2 ± 0.6	35.2 ± 0.2	25.9 ± 0.2	35.2 ± 0.2	25.9 ± 0.2	35.2 ± 0.5	51.7 ± 0.5	35.2 ± 0.2
	**MF** **C**	70.5 ± 0.6	70.5 ± 0.4	35.2 ± 0.4	70.5 ± 0.8	35.2 ± 0.2	70.5 ± 0.5	70.5 ± 0.5	70.5 ± 0.5
**5c**	**MIC**	282.0 ± .2.0	35.3 ± 0.2	18.8 ± 0.2	25.9 ± 0.1	18.8 ± 0.2	35.3 ± 0.2	35.3 ± 0.2	35.3 ± 0.2
	**MFC**	564.1 ± 4.0	68.2 ± 0.4	35.3 ± 0.2	35.3 ± 0.2	35.3 ± 0.5	68.2 ± 0.5	68.2 ± 0.5	68.2 ± 0.5
**5d**	**MIC**	202 ± 0.1	37.9 ± 0.2	27.8 ± 0.1	37.9 ± 0.0	27.8 ± 0.2	37.9 ± 0.5	37.9 ± 0.2	37.9 ± 0.2
	**MFC**	37.9 ± 0.2	75.6 ± 0.4	37.9 ± 0.5	75.6 ± 0.5	37.9 ± 0.5	75.6 ± 0.5	75.6 ± 0.5	75.6 ± 0.5
**5e**	**MIC**	39.4 ± 0.2	39.4 ± 0.2	21.0 ± 0.1	39.4 ± 0.5	21.0 ± 0.1	39.4 ± 0.5	39.4 ± 0.5	39.4 ± 0.5
	**MFC**	78.9 ± 0.4	78.9 ± 0.4	39.4 ± 0.2	78.9 ± 1.0	39.4 ± 0.5	78.9 ± 0.5	78.9 ± 0.5	78.9 ± 0.5
**5f**	**MIC**	76.1 ± 0.4	38.0 ± 0.2	27.9 ± 0.1	38.0 ± 0.5	27.9 ± 0.2	55.8 ± 0.5	55.8 ± 0.5	55.8 ± 0.5
	**MFC**	152.1 ± 0.1	76.1 ± 0.4	38.0 ± 0.5	76.1 ± 0.8	38.0 ± 0.5	76.1 ± 1.0	76.1 ± 0.8	76.1 ± 1.0
**5g**	**MIC**	73.1 ± 0.4	19.5 ± 0.2	19.5 ± 0.1	14.6 ± 0.1	9.7 ± 0.01	36.5 ± 0.5	19.5 ± 0.1	26.8 ± 0.1
	**MFC**	146.2 ± 0.1	36.5 ± 0.4	36.5 ± 0.5	19.5 ± 0.1	19.5 ± 0.08	73.1 ± 0.8	36.5 ± 0.5	36.5 ± 0.2
**5h**	**MIC**	315.5 ± 2.5	78.9 ± 1.0	39.4 ± 0.2	78.9 ± 0.5	28.9 ± 0.1	39.4 ± 0.5	78.9 ± 0.5	118.3 ± 1.0
	**MFC**	630.9 ± 8.0	157.7 ± 1.0	78.9 ± 0.8	157.7 ± 1.0	39.4 ± 0.2	78.9 ± 0.5	157.7 ± 1.0	157.7 ± 2.0
**5i**	**MIC**	152.1 ± 1.0	38.0 ± 0.4	38.0 ± 0.0	38.0 ± 0.0	27.9 ± 0.2	76.1 ± 0.5	55.8 ± 0.5	76.1 ± 0.5
	**MFC**	304.2 ± .2.0	76.1 ± 0.0	76.1 ± 1.0	76.1 ± 0.8	38.0 ± 0.5	152.1 ± 1.0	76.1 ± 0.8	152.1 ± 1.0
**5j**	**MIC**	146.2 ± 1.0	35.5 ± 0.2	53.6 ± 0.4	26.8 ± 0.2	35.5 ± 0.5	35.5 ± 0.4	35.5 ± 0.4	35.5 ± 0.6
	**MFC**	292.3 ± .2.0	73.1 ± 0.8	73.1 ± 0.8	35.5 ± 0.5	73.1 ± 1.0	73.1 ± 1.0	73.1 ± 1.0	73.1 ± 1.0
**5k**	**MIC**	35.5 ± 0.4	35.5 ± 0.2	35.5 ± 0.2	35.5 ± 0.5	35.5 ± 0.2	35.5 ± 0.5	35.5 ± 0.4	35.5 ± 0.2
	**MFC**	73.1 ± 0.8	73.1 ± 0.8	73.1 ± 1.0	73.1 ± 0.5	73.1 ± 0.8	73.1 ± 0.8	73.1 ± 0.5	73.1 ± 0.8
**7a**	**MIC**	347.4 ± 2.0	43.4 ± 0.2	43.4 ± 0.2	43.4 ± 0.2	31.8 ± 0.2	43.4 ± 0.2	43.4 ± 0.4	86.9 ± 1.0
	**MFC**	694.8 ± 4.0	86.9 ± 1.0	86.9 ± 0.5	86.9 ± 1.0	43.4 ± 0.5	86.9 ± 1.0	86.9 ± 1.0	173.7 ± 2.0
**7b**	**MIC**	22.3 ± 0.1	22.3 ± 0.1	22.3 ± 0.1	41.7 ± 0.5	22.3 ± 0.2	41.7 ± 0.5	41.7 ± 1.0	22.3 ± 0.1
	**MFC**	41.7 ± 0.2	41.7 ± 0.2	41.7 ± 0.5	83.4 ± .1.0	41.7 ± 0.8	83.4 ± 1.0	83.4 ± 1.0	41.7 ± 0.4
**7c**	**MIC**	79.9 ± 0.4	21.3 ± 0.1	16.0 ± 0.1	21.3 ± 0.2	10.7 ± 0.2	21.3 ± 0.1	21.3 ± 1.0	58.6 ± 0.4
	**MFC**	159.8 ± 1.0	39.9 ± 0.5	21.3 ± 0.1	39.9 ± 0.2	21.3 ± 0.5	39.9 ± 0.2	39.9 ± 1.0	79.9 ± 1.0
**Ket.**	**MIC**	380 ± 12	2850 ± 68	380 ± 12	380 ± 8.0	475 ± 58	3800 ± 58	380 ± 16	380 ± 12
	**MFC**	950 ± 23	3800 ± 84	950 ± 12	950 ± 6.0	570 ± 86	3800 ± 48	950 ± 26	950 ± 23
**Bif.**	**MIC**	480 ± 22	480 ± .2	480 ± 28	480 ± 12	640 ± 28	480 ± 20	640 ± 12	480 ± 22
	**MFC**	640 ± 3.4	640 ± 0.8	800 ± 1.8	640 ± 2.3	800 ± 3.8	640 ± 1.6	800 ± 2.1	640 ± 3.4

MIC–minimal inhibitory concentration, MFC–minimal fungicidal concentration*. A.fum.-A.fumigatus* (human isolate), *A.v.-A.versicolor* (ATCC 11730), *A.o.-A.ochraceus* (ATCC 12066), *A.n.-A.niger* (ATCC 6275), *T.v.-T.viride* (IAM 5061), *P.f.-P.funiculosum* (ATCC 36839), *P.o.-P.ochrochloron* (ATCC 9112), *P.v.c.-P.cyclpoium var. verucosum* (food isolate).

**Table 5:** Antibacterial activity (MIC), cytotoxicity (CC_50_), and selectivity indices (SI) of compounds **5d, 5g, 5k, 7c.**

ID	CC_50_		*B.c*	*M.f*	*S.a*	*L.m*	*En.cl*	*P.a*	*S.T*	*E.coli*
**5d**		**MIC**	55.6 ± 0.2	113.8 ± 0.8	37.9 ± 0.2	113.8 ± 0.8	37.9 ± 0.4	37.9 ± 0.2	113.8 ± 1.0	75.6 ± 0.4
	252 ± 1.5	**SI**	4.5	2.2	6.7	2.2	6.7	6.7	2.2	3.3
**5g**		**MIC**	73.1 ± 0.1	73.1 ± 1.0	53.6 ± 0.4	73.1 ± 0.8	36.5 ± 0.5	109.6 ± 1.0	53.6 ± 0.6	109.6 ± 1.0
	256 ± 6.21	**SI**	3.5	3.5	4.8	3.5	7.0	2.3	4.8	2.3
**5k**		**MIC**	73.1 ± 0.5	109.6 ± 1.0	58.6 ± 0.4	109.6 ± 1.5	58.6 ± 0.6	58.6 ± 0.6	109.6 ± 1.5	109.6 ± 2.0
	252 ± 1.89	**SI**	3.5	2.3	4.3	2.3	4.3	4.3	2.3	2.3
**7c**		**MIC**	79.9 ± 0.4	119.8 ± 1.5	79.9 ± 1.0	159.8 ± 1.0	58.6 ± 0.4	58.6 ± 0.4	119.8 ± 1.0	119.8 ± 1.5
	225± 1.87	**SI**	2.8	1.9	2.8	1.4	3.8	3.8	1.9	1.9

**Table 6:** Antifungal activity (MIC), cytotoxicity (CC_50_), and selectivity indices (SI) of compounds **5d, 5g, 5k, 7c.**

Com.	CC_50_ (μM)		*A.f*	*A.v*	*A.o*	*A.n*	*T.v*	*P.o*	*P.f*	*Pvc*
**5d**		**MIC**	202 ± 0.1	37.9 ± 0.2	27.8 ± 0.1	37.9 ± 0.2	27.8 ± 0.2	37.9 ± 0.5	37.9 ± 0.2	37.9 ± 0.2
	252 ± 1.5	**SI**	1.3	6.7	9.1	6.7	9.1	6.7	6.7	6.7
**5g**		**MIC**	73.1 ± 0.2	19.5 ± 0.2	19.5 ± 0.2	14.6 ± 0.1	9.7 ± 0.1	36.5 ± 0.5	19.5 ± 0.1	26.8 ± 0.1
	256 ± 6.21	**SI**	3.5	13.1	13.1	17.5	26.4	7.0	13.1	9.6
**5k**		**MIC**	35.5 ± 0.4	35.5 ± 0.2	35.5 ± 0.2	35.5 ± 0.5	35.5 ± 0.2	35.5 ± 0.5	35.5 ± 0.4	35.5 ± 0.2
	252 ± 1.89	**SI**	7.1	7.1	7.1	7.1	7.1	7.1	7.1	7.1
**7c**		**MIC**	79.9 ± 0.4	21.3 ± 0.1	16.0 ± 0.1	21.3 ± 0.2	10.7 ± 0.2	21.3 ± 0.1	21.3 ± 1.0	58.6 ± 0.4
	225 ± 1.87	**SI**	2.8	10.6	14.1	10.6	21.0	10.6	10.6	3.8
**Ket.**		**MIC**	380 ± 12	285 ± 68	380 ± 12	380 ± 8.0	475 ± 58	380 ± 58	380 ± 16	380 ± 12
	60 *	**SI**	0.158	0.210	0.158	0.158	0.126	0.158	0.158	0.158

* 24 h.

**Table 7:** Molecular docking binding energies.

Comp.	Est. Binding Energy (kcal/mol)			I-H *E. coli MurB*	Residues *E. coli MurB*
	* E.coli* *DNA Gyrase* *1KZN *	*Thymidylate Kinase* *4QGG*	*E. coli MurB 2Q85*		
**5a**	−4.63	-	−8.22	2	Gly122, Ser228
**5b**	−3.12	-	−7.70	2	Arg213, Asn232
**5c**	−5.39	−2.13	-9.16	2	Gly122, Ser228
**5d**	−6.21	−4.12	−10.93	2	Ser228, Arg326
**5e**	−6.28	−2.39	−8.97	2	Arg213, Ser228
**5f**	−5.46	−1.55	−8.74	2	Gly122, Ser228
**5g**	−6.54	−3.26	−10.88	3	Gly122, Ser228, Asn232
**5h**	−6.11	−1.24	−9.12	2	Gly122, Ser228
**5i**	−3.69	−1.15	−7.07	2	Arg213, Arg326
**5j**	−5.52	−3.25	−9.21	2	Gly122, Ser228
**5k**	−5.63	−2.96	−9.83	2	Arg213, Ser228
**7a**	−2.59	-	−7.28	2	Gly122, Arg213
**7b**	−3.67	-	−7.75	2	Ser228, Asn232
**7c**	−4.28	-	−7.88	2	Gly122, Ser228

**Table 8:** Molecular docking binding energies.

N/N	Est. Binding Energy (kcal/mol) CYP51 of *C. albicans* PDB ID: 5V5Z	I-H	Residues CYP51 of *C. albicans* PDB ID: 5V5Z	Interactions with HEM601
**5a**	−7.65	1	Tyr132	Hydrophobic
**5b**	−9.74	1	Tyr132	Ionizable, Hydrophobic
**5c**	−8.13	2	Tyr64, Tyr132	Hydrophobic
**5d**	−10.22	2	Tyr118, Tyr132	Ionizable, Hydrophobic
**5e**	−9.15	1	Tyr132	Ionizable, Hydrophobic
**5f**	−8.79	2	Tyr118, Tyr132	Hydrophobic
**5g**	−11.55	1	Tyr132	Fe binding, Ionizable, Hydrophobic
**5h**	−7.11	-	-	Ionizable, Hydrophobic
**5i**	−7.84	1	Tyr132	Ionizable, Hydrophobic
**5j**	−8.72	2	Tyr118, Met508	Hydrophobic
**5k**	−9.24	2	Tyr64, Tyr118	Hydrophobic
**7a**	−7.08	1	Tyr132	Hydrophobic
**7b**	−10.36	1	Tyr132	Ionizable, Hydrophobic
**7c**	−10.84	2	Tyr118, Tyr132	Ionizable, Hydrophobic
**ketoconazole**	−8.23	1	Tyr64	Ionizable, Hydrophobic

## Supplementary Materials

The following are available online at https://www.mdpi.com/1424-8247/13/9/229/s1, Supplementary file PASSweb_results_13mols.xlsx: Predictions of antimicrobial activity and acute rat toxicity.

## References

[1] Michaud C.M. (2009). Global Burden of Infectious Diseases. Encycl. Microbiol..

[2] Nii-Trebi N.I. (2017). Emerging and neglected infectious diseases: Insights, advances, and challenges. Biomed. Res. Int..

[3] Mukherjee S. (2017). Emerging Infectious Diseases: Epidemiological Perspective. Indian J. Dermatol..

[4] Ventola C.L. (2015). The antibiotic resistance crisis: Part 1: Causes and threats. Pharm. Ther..

[5] Michael C.A., Dominey-Howes D., Labbate M. (2014). The antimicrobial resistance crisis: Causes, consequences, and management. Front. Public Health.

[6] Rice L.B. (2008). Federal funding for the study of antimicrobial resistance in nosocomial pathogens: No ESKAPE. J. Infect. Dis..

[7] Holmes A.H., Moore L.S., Sundsfjord A., Steinbakk M., Regmi S., Karkey A., Guerin P.J., Piddock L.J. (2016). Understanding the mechanisms and drivers of antimicrobial resistance. Lancet.

[8] Tripathi A.C., Gupta S.J., Fatima G.N., Sonar P.K., Verma A., Saraf S.K. (2014). 4- Thiazolidinones: The Advances Continue…. Eur. J. Med. Chem..

[9] Kaminskyy D., Kryshchyshyn A., Lesyk R. (2017). 5-Ene-4-thiazolidinones–An efficient tool in medicinal chemistry. Eur. J. Med. Chem..

[10] Kaminskyy D., Kryshchyshyn A., Lesyk R. (2017). Recent developments with rhodanine as a scaffold for drug discovery. Expert Opin. Drug Discov..

[11] Baell B. (2010). Observations on screening-based research and some concerning trends in the literature. Future Med. Chem..

[12] Mendgen T., Steuer C., Klein C.D. (2012). Privileged scaffolds or promiscuous binders: A comparative study on rhodanines and related heterocycles in medicinal chemistry. J. Med. Chem..

[13] Morphy R., Rankovic Z. (2005). Designed multiple ligands. An emerging drug discovery paradigm. J. Med. Chem..

[14] Fortin S., Bérubé G. (2013). Advances in the development of hybrid anticancer drugs. Expert Opin. Drug Discov..

[15] Kryshchyshyn A., Roman O., Lozynskyi A., Lesyk R. (2018). Thiopyrano[2,3-d]thiazoles as new efficient scaffolds in medicinal chemistry. Sci. Pharm..

[16] Cong N.T., Nhan H.T., Van Hung L., Thang T.D., Kuo P.C. (2014). Synthesis and antibacterial activity of analogs of 5-arylidene-3-(4-methylcoumarin-7-yloxyacetylamino)-2-thioxo-1,3-thiazoli-din-4-one. Molecules.

[17] Song M.-X., Deng X.-Q., Li Y.-R., Zheng C.-J., Hong L., Piao H.-R. (2014). Synthesis and biological evaluation of (E)-1-(substituted)-3-phenylprop-2-en-1-ones bearing rhodanines as potent anti-microbial agents. J. Enzyme Inhib. Med. Chem..

[18] Krátký M., Vinšová J., Stolaříková J. (2017). Antimicrobial activity of rhodanine-3-acetic acid derivatives. Bioorg. Med. Chem..

[19] Horishny V., Kartsev V., Geronikaki A., Matiychuk V., Petrou A., Glamoclija J., Ciric A., Sokovic M. (2020). 5-(1H-Indol-3-ylmethylene)-4-oxo-2-thioxothiazolidin-3-yl)alkancarboxylic Acids as Antimicrobial Agents: Synthesis, Biological Evaluation, and Molecular Docking Studies. Molecules.

[20] Incerti M., Vicini P., Geronikaki A., Eleftheriou P., Tsagkadouras A., Zoumpoulakis P., Fotakis C., Ćirić A., Glamočlija J., Soković M. (2017). New N-(2-phenyl-4-oxo-1,3-thiazolidin-3-yl)-1,2-benzothiazole -3-carboxamides and Acetamides as Antimicrobial Agents. Med. Chem. Commun..

[21] Ozen C., Ceylan-Unlusoy M., Aliary N., Ozturk M., Bozdag-Dundar O. (2017). Thiazolidinedione or Rhodanine: A Study on Synthesis and Anticancer Activity Comparison of Novel Thiazole Derivatives. Pharm. Pharm. Sci..

[22] Fu H., Hou X., Wang L., Dun Y., Yang X., Fang H. (2015). Design, synthesis and biological evaluation of 3-aryl-rhodanine benzoic acids as anti-apoptotic protein Bcl-2 inhibitors. Bioorg. Med. Chem. Lett..

[23] Havrylyuk D., Zimenkovsky B., Lesyk R. (2009). Synthesis and Anticancer Activity of Novel Nonfused Bicyclic Thiazolidinone Derivatives. Phosphorus Sulfur.

[24] El-Miligy M., Hazzaa A., El-Messmary H., Nassra R.A. (2017). El-Hawash, Soad, New hybrid molecules combining benzothiophene or benzofuran with rhodanine as dual COX-1/2 and 5-LOX inhibitors: Synthesis, biological evaluation and docking study. Bioorg. Chem..

[25] R. Atta-Allah S., Nassar I.F., El-Sayed W.A. (2020). Design, synthesis and anti-inflammatoryvel 5-(Indol-3-yl)-thiazolidinone derivatives as COX-2 inhibitors. J. Pharmacol. Ther. Res..

[26] Powers J.P., Piper D.E., Li Y., Mayorga V., Anzola J., Chen J.M., Jaen J.C., Lee G., Liu J., Peterson M.G. (2006). SAR and mode of action of novel non-nucleoside inhibitors of hepatitis C NS5b RNA polymerase. J. Med. Chem..

[27] Ramkumar K., Yarovenko V.N., Nikitina A.S., Zavarzin I.V., Krayushkin M.M., Kovalenko L.V., Esqueda A., Odde S., Neamati N. (2010). Design, synthesis and structure-activity studies of rhodanine derivatives as HIV-1 integrase inhibitors. Molecules.

[28] Petrou A., Eleftheriou P., Geronikaki A., Akrivou M.G., Vizirianakis I. (2019). Novel thiazolidin-4-ones as potential non-nucleoside inhibitors of HIV-1 reverse transcriptase. Molecules.

[29] Kryshchyshyn A., Kaminskyy D., Roman O., Kralovics R., Karpenko O., Lesyk R. (2020). Synthesis and anti-leukemic activity of pyrrolidinedione-thiazolidinone hybrids. Ukr Biochem. J..

[30] Havrylyuk D., Zimenkovsky B., Vasylenko O., Gzella A., Lesyk R. (2012). Synthesis of new 4- thiazolidinone-, pyrazoline-, and isatin-based conjugates with promising antitumor activity. J. Med. Chem..

[31] Havrylyuk D., Roman O., Lesyk R. (2016). Synthetic approaches, structure activity relationship and biological applications for pharmacologically attractive pyrazole/pyrazoline–thiazolidine– based hybrids. Eur. J. Med. Chem..

[32] Saini T., Kumar S., Narasimhan B. (2016). Central nervous system activities of indole derivatives: An overview. Cent. Nerv. Syst. Agents Med. Chem..

[33] Singh P., Singh O.M. (2018). Recent progress in biological activities of indole and indole alkaloids. Mini Rev. Med. Chem..

[34] Kaur J., Utreja D., Jain N., Sharma S. (2019). Recent Developments in the Synthesis and Antimicrobial Activity of Indole and Its Derivatives. Curr. Org. Synth..

[35] Bathula C., Tripathi S., Srinivasan R., Jha K.K., Ganguli A., Chakrabarti G., Singh S., Munshi P., Sen S. (2016). Synthesis of novel 5-arylidenethiazolidinones with apoptotic properties via a three component reaction using piperidine as a bifunctional reagent. Org. Biomol. Chem..

[36] Sayed M., El-Dean A., Ahmed M., Hassanien R. (2018). Synthesis of some heterocyclic compounds derived from indole as antimicrobial agents. Synth. Commun..

[37] Jain P., Utreja D., Sharma P. (2020). An efficacious synthesis of N-1–, C-3–substituted indole derivatives and their antimicrobial studies. J. Hetrocyclic Chem..

[38] Shaikh T.M.A., Debebe H. (2020). Synthesis and Evaluation of Antimicrobial Activities of Novel N-Substituted Indole Derivatives. J. Chem..

[39] Kumar P., Singh S., Rizki M., Pratama F. (2020). Synthesis of some novel 1H-indole derivatives with antibacterial activity and antifungal activity. Lett. Appl. NanoBioScience.

[40] Liu Z., Tang L., Zhu H., Xu T., Qiu C., Zheng S., Gu Y., Feng J., Zhang Y., Liang G. (2016). Design, Synthesis, and Structure−Activity Relationship Study of Novel Indole-2-carboxamide Derivatives as Anti-inflammatoryAgents for the Treatment of Sepsis. J. Med. Chem..

[41] Li S., Wang Z., Xiao H., Bian Z., Wang J. (2020). Enantioselective synthesis of indole derivatives byRh/Pd relay catalysis and their anti-inflammatory evaluation. Chem. Commun..

[42] Abdellatif K.R.A., Elsaady M.Y., Amin N.H., Hefny A.A. (2017). Design, Synthesis and biological evaluation of some novel indole derivatives as selective COX-2 inhibitors. J. Appl. Pharm. Sci..

[43] Bhat M.A., Al-Omar M.A., Raish M. (2018). Indole Derivatives as Cyclooxygenase Inhibitors: Synthesis, Biological Evaluation and Docking Studies. Molecules.

[44] Sidhu J.S., Singla R., Jaitak V. (2015). Indole Derivatives as Anticancer Agents for Breast Cancer Therapy: A Review. Anticancer Agents Med. Chem..

[45] El-Sharief A.M.S., Ammar Y.A., Belal A., El-Sharief M.A.S., Mohamed Y.A., Ahmed B.M., Mehany A.B.M., Elhag Ali G.A.M., Ragab A. (2019). Design, synthesis, molecular docking and biological activity evaluation of some novel indole derivatives as potent anticancer active agents and apoptosis inducers. Bioorg. Chem..

[46] Cascioferro S., Li Petri G., Parrino B., El Hassouni B., Carbone D., Arizza V., Perricone U., Padova A., Funel N., Peters G.J. (2020). 3-(6-Phenylimidazo [2,1-b][1,3,4]thiadiazol-2-yl)-1H-Indole Derivatives as New Anticancer Agents in the Treatment of Pancreatic Ductal Adenocarcinoma. Molecules.

[47] Zhang M.-Z., Chen Q., Yang G.F. (2015). A review on recent developments of indole-containing antiviral agents. J. Med. Chem..

[48] Bardiot D. (2018). Discovery of Indole Derivatives as Novel and Potent Dengue Virus Inhibitors. J. Med. Chem..

[49] Che Ζ., Tian Υ., Liu S., Hu M., Che G. (2019). Discovery of N-arylsulfonyl-3-acylindole benzoyl hydrazone derivatives as anti-HIV-1 agents. Braz. J. Pharm. Sci.

[50] Sanna G., Madeddu S., Giliberti G., Piras S., Struga M., Wrzosek M., Kubiak-Tomaszewska G., Koziol A.E., Savchenko O., Lis T. (2018). Synthesis and Biological Evaluation of Novel Indole-Derived Thioureas. Molecules.

[51] Ramya V., Vembu S., Ariharasivakumar G., Gopalakrishnan M. (2017). Synthesis, Characterisation, Molecular Docking, Anti-microbial and Anti-diabetic Screening of Substituted 4-indolylphenyl-6-arylpyrimidine-2-imine Derivatives. Drug Res. (Stuttg).

[52] Tymiak A.A., Rinehart K.L., Bakus G.J. (1985). Constituents of morphologically similar sponges: Aplysina and Smenospongia species. Tetrahedron.

[53] Djura P., Stierle D.B., Sullivan B. (1980). Some metabolites of the marine sponges Smenospongia aurea and Smenospongia (ident.Polyfibrospongia) echina. J. Org.Chem..

[54] Fattorusso E., Lanzotti V., Magno S., Novellino E. (1985). Tryptophan derivatives from a Mediterranean anthozoan, Astroides calycularis. J. Nat. Prod..

[55] Buyukbingol E., Suzen S., Klopman G. (1994). Studies on the synthesis and structure–activity relationships of 5-(3′-indolyl)-2-thiohydantoin derivatives as aldose reductase enzyme inhibitors. Farmaco.

[56] Pogodin P.V., Lagunin A.A., Rudik A.V., Druzhilovskiy D.S., Filimonov D.A., Poroikov V.V. (2019). AntiBac-Pred: A Web Application for Predicting Antibacterial Activity of Chemical Compounds. J. Chem. Inf. Model..

[57] Poroikov V., Filimonov D., Gloriozova T., Lagunin A., Druzhilovskiy D., Rudik A., Stolbov L., Dmitriev A., Tarasova O., Ivanov S. (2019). Computer-aided prediction of biological activity spectra for organic compounds: The possibilities and limitations. Russ. Chem. Bull..

[58] Clarivate Analytics Integrity. https://integrity.clarivate.com/integrity/.

[59] Antifungal Activity Predictor. http://www.way2drug.com/micf.

[60] Filimonov D.A., Zakharov A.V., Lagunin A.A., Poroikov V.V. (2009). QNA-based ‘Star Track’ QSAR approach. SAR QSAR Env. Res..

[61] Lagunin A., Zakharov A., Filimonov D., Poroikov V. (2011). QSAR Modelling of Rat Acute Toxicity on the Basis of PASS Prediction. Mol. Inform..

[62] Bruno G., Costantino L., Curinga C., Maccari R., Monforte F., Nicolo F., Ottana R., Vigorita M.G. (2002). Synthesis and Aldose Reductase Inhibitory Activity of5-Arylidene-2,4-thiazolidinediones. Bioorg. Med. Chem..

[63] Abdeen S., Kunkle T., Salim N., Ray A.-M., Mammadova N., Summers C., Stevens M., Ambrose A.J., Park Y., Schultz P.G. (2018). Sulfonamido-2-arylbenzoxazole GroEL/ES Inhibitors as Potent Antibacterials against Methicillin-Resistant Staphylococcus aureus (MRSA). J. Med. Chem..

[64] Menozzi G., Merello L., Fossa P., Ranise A., Mosti L., Bondavalli F., Loddo R., Murgioni C., Mascia V., La Colla P. (2004). Synthesis, antimicrobial activity and molecular modeling studies of halogenated 4-[1H-imidazol-1-yl(phenyl)methyl]-1,5-diphenyl-1H-pyrazoles. Bioorg. Med. Chem..

[65] Vieira F.T., de Lima G.M., Maia J.R., Speziali N.L., Ardisson J.D., Rodrigues L., Correa A., Romero O.B. (2010). Synthesis, characterization and biocidal activity of new organotin complexes of 2-(3-oxocyclohex-1-enyl)benzoic acid. Eur. J. Med. Chem..

[66] Gjorgjieva M., Tomasicč T., Barancokova M., Katsamakas S., Ilas J., Tammela P., Masicč L.P., Kikelj D. (2016). Discovery of Benzothiazole Scaffold-Based DNA Gyrase B Inhibitors. J. Med. Chem..

[67] Parvathy N.G., Manju P., Mukesh M., Thomas L. (2013). Design, synthesis and molecular docking studies of benzothiazole derivatives as anti microbial agents. Int. J. Pharm. Pharm. Sci..

[68] Ren Y., Zhang L., Zhou C.H., Geng R.X. (2014). Recent Development of Benzotriazole-based Medicinal Drugs. Med. Chem..

[69] Andres C.J., Bronson J.J., D’Andrea S.V., Walsh A.W. (2000). 4-thiazolidinones: Novel inhibitors of the bacterial enzyme MurB. Bioorg. Med. Chem. Lett..

[70] Ahmed S., Zayed M.F., El-Messery S.M., Al-Agamy M.H., Abdel-Rahman H.M. (2016). Design, Synthesis, Antimicrobial Evaluation and Molecular Modeling Study of 1,2,4-Triazole-Based 4-Thiazolidinones. Molecules.

[71] Pitta E., Tsolaki E., Geronikaki A., Petrovic J., Glamočlija J., Sokovic M., Crespan E., Maga G., Bhunia S.S., Saxena A.K. (2015). 4-Thiazolidinone derivatives as potent antimicrobial agents: Microwave-assisted synthesis, biological evaluation and docking studies. MedChemComm.

[72] Karanth S., Narayana B., Kodandoor S.C., Sarojini B.K. (2018). 2-{[(4-Hydroxy-3,5-dimethoxyphenyl)methylidene]hydrazinylidene}-4-oxo-1,3-thiazolidin-5-yl Acetic Acid. Molbank.

[73] Stana A., Vodnar D.C., Tamaian R., Pîrnău A., Vlase L., Ionuț I., Oniga O., Tiperciuc B. (2017). Design, Synthesis and Antifungal Activity Evaluation of New Thiazolin-4-ones as Potential Lanosterol 14α-Demethylase Inhibitors. Int. J. Mol. Sci..

[74] Incerti M., Vicini P., Geronikaki A., Eleftheriou P., Tsagkadouras A., Zoumpoulakis P., Fotakis C., Ćirić A., Glamočlija J., Soković M. (2017). New N-(2-phenyl-4-oxo-1,3-thiazolidin-3-yl)-1,2-benzothiazole-3-carboxamides and acetamides asantimicrobial agents. Med. Chem. Commun..

[75] Can N.O., Çevik U.A., Sağlık B.N., Levent S., Korkut B., Özkay Y., Kaplancıklı Z.A., Koparal A.S. (2017). Synthesis, Molecular Docking Studies, and Antifungal Activity Evaluation of New Benzimidazole-Triazoles as Potential Lanosterol 14*α*-Demethylase Inhibitors. J. Chem..

[76] Benson T.E., Walsh C.T., Massey V. (1997). Kinetic characterization of wild-type and S229A mutant MurB: Evidence for the role of Ser 229 as a general acid. Biochemistry.

[77] Kartsev V., Lichitsky B., Geronikaki A., Petrou A., Smiljkovic M., Kostic M., Radanovic O., Soković M. (2018). Design, synthesis and antimicrobial activity of usnic acid derivatives. MedChemComm.

[78] Fesatidou M., Zagaliotis P., Camoutsis C., Petrou A., Eleftheriou P., Tratrtat C., Haroun M., Geronikaki A., Ciric A., Sokovic M. (2018). 5-Adamantan thiadiazole-based thiazolidinones as antimicrobial agents. Design, synthesis, molecular docking and evaluation. Bioorg. Med. Chem..

[79] Kostić M., Smiljković M., Petrović J., Glamočilija J., Barros L., Ferreira I.C.F.R., Ćirić A., Soković M. (2017). Chemical, nutritive composition and a wide range of bioactive properties of honey mushroom Armillaria mellea (Vahl: Fr.) Kummer. Food Function.

[80] Kritsi E., Matsoukas M.T., Potamitis C., Detsi A., Ivanov M., Sokovic M., Zoumpoulakis P. (2019). Novel Hit Compounds as Putative Antifungals: The Case of Aspergillus fumigatus. Molecules.

[81] Aleksić M., Stanisavljević D., Smiljković M., Vasiljević P., Stevanović M., Soković M., Stojković D. (2019). Pyrimethanil: Between efficient fungicide against Aspergillus rot on cherry tomato and cytotoxic agent on human cell lines. Ann. App. Bio..

